# Risk thresholds for soft versus hard cardiovascular disease outcome models for initiating statin therapy among Chinese adults: a cost-utility analysis

**DOI:** 10.1186/s12916-025-04222-8

**Published:** 2025-07-01

**Authors:** Zhijia Sun, Haijun Zhang, Yinqi Ding, Canqing Yu, Dianjianyi Sun, Yuanjie Pang, Pei Pei, Ling Yang, Yiping Chen, Huaidong Du, Dan Huang, Xiaoming Yang, Maxim Barnard, Robert Clarke, Junshi Chen, Zhengming Chen, Liming Li, Jun Lv, on behalf of the China Kadoorie Biobank Collaborative Group

**Affiliations:** 1 Department of Epidemiology & Biostatistics, School of Public Health, Peking University, Beijing, 100191 China; 2 Department of Health Policy and Management, School of Public Health, Peking University, Beijing, 100191 China; 3 Department of International Health, Johns Hopkins Bloomberg School of Public Health, Baltimore, 21205 USA; 4 Center for Public Health and Epidemic Preparedness & Response, Peking University, Beijing, 100191 China; 5 Key Laboratory of Epidemiology of Major Diseases (Peking University), Ministry of Education, Beijing, China; 6 Clinical Trial Service Unit & Epidemiological Studies Unit (CTSU), Nuffield Department of Population Health, University of Oxford, Oxford, UK; 7 Record Department, Pengzhou Traditional Chinese Medical Hospital, Sichuan, 611930 China; 8 China National Center for Food Safety Risk Assessment, Beijing, China; 9 State Key Laboratory of Vascular Homeostasis and Remodeling, Peking University, Beijing, China

**Keywords:** Cardiovascular disease, Cost-utility analysis, Primary prevention, Hydroxymethylglutaryl-CoA reductase inhibitors, China

## Abstract

**Background:**

Current guidelines for atherosclerotic cardiovascular disease (ASCVD) primary prevention mostly recommend risk scores that predict risk of non-fatal myocardial infarction, fatal ischemic heart disease (IHD), and fatal or non-fatal ischemic stroke (hard outcomes), ignoring the burden from other non-fatal IHD outcomes. We explored the optimal risk thresholds for statin initiation using non-laboratory-based soft and hard ASCVD outcome models and compared the cost-utility of such models in the Chinese population.

**Methods:**

We constructed Markov cohort models to estimate the incidence of ASCVD events, costs, and quality-adjusted life years (QALYs) over a lifetime from a social perspective. The simulation cohort was constructed using data from the China Kadoorie Biobank (CKB). Input data included cost, utility, statin efficacy, and other parameters were derived from published literature. We used CKB-ASCVD models to predict 10-year risk and different risk thresholds to guide statin initiation. The incremental cost-effectiveness ratio (ICER) was estimated as cost per QALY gained. Sensitivity analyses were performed to explore the uncertainty in the models.

**Results:**

The optimal risk threshold was 18% for the soft ASCVD model and 10% for the hard ASCVD model, with ICERs of $7013.48/QALY and $6540.71/QALY, respectively. The optimal thresholds were robust in stratified analyses by region and sex, and one-way sensitivity analyses over a wide range of input parameters. Probabilistic sensitivity analyses showed that these optimal thresholds had around 70% chance of being cost-effective. When analyzed by age group, above optimal thresholds were cost-effective in adults aged 30–59 years but not in those aged 60–75 years. The threshold strategies based on soft ASCVD model were mostly cost-saving compared with those based on hard models to treat the same proportions of the population.

**Conclusions:**

The risk threshold of 18% for soft ASCVD model and 10% for hard ASCVD model have acceptable cost-utility profiles in the Chinese population. The soft ASCVD model is more cost-effective than the hard model and should be used as a screening tool for ASCVD primary prevention.

**Supplementary Information:**

The online version contains supplementary material available at 10.1186/s12916-025-04222-8.

## Background

Atherosclerotic cardiovascular disease (ASCVD) is mainly composed of ischemic heart disease (IHD) and ischemic stroke (IS) and is the leading cause of death and disability worldwide [1]. There has been a continuous increase in the morbidity and mortality of ASCVD over the past three decades in China [2, 3]. From 1990 to 2015, although the age-standardized years of life lost (YLLs) from IHD declined in China, the age-standardized years lived with disability (YLDs) from IHD increased by 23.8%, highlighting the importance of targeting loss of healthy lifespan from non-fatal IHD events [4].

Current clinical guidelines recommend the use of quantitative 10-year ASCVD risk scores tools to identify high-risk individuals and guide statin initiation [5–11]. The recommended risk thresholds for statin therapy vary from 5 to 12% [12] (e.g., the American College of Cardiology and American Heart Association [ACC/AHA] use 7.5% [5]). The risk thresholds in some guidelines were supported by evidence from cost-utility analyses [13–15], which are especially important for establishing their affordability at national and local levels, given the available resources. A previous study reported that the availability of low-cost generic forms of statins and the implementation of China’s centralized medicine procurement policy have made statin prices affordable and statin therapy cost-effective for primary prevention [16]. Risk thresholds of 10% for the prediction equations used in Chinese guidelines have been evaluated as being cost-effective when using the willingness to pay (WTP) of three times the gross domestic product (GDP) per capita [17].

All existing guidelines, except for those from the UK and New Zealand [9, 10], use hard ASCVD outcomes (including non-fatal myocardial infarction [MI], fatal IHD, and fatal or non-fatal IS) as endpoints of risk prediction models. In contrast, soft ASCVD outcomes refer to non-fatal IHD events other than MI, plus hard ASCVD outcomes. A study based on hospitalization and death data in New Zealand reported that about 30% of total IHD hospitalizations and deaths were non-fatal non-MI IHD events [18]. A study using the long-term follow-up data of China Kadoorie Biobank (CKB) found that non-fatal non-MI IHD events accounted for about 80% of the total IHD events [19]. Therefore, using prediction models with hard ASCVD outcomes to define high-risk populations could result in under treatment of some individuals at high risk for non-fatal IHD. These individuals contribute to a considerable amount of non-fatal IHD events and costs and disability due to hospitalization and chronic treatment. Whether there is a difference in the cost-utility of using the risk prediction models with hard and soft ASCVD outcomes (hereafter referred to as hard and soft ASCVD models) as screening tools for primary prevention has not been previously evaluated.

Using data from CKB, a large prospective cohort of Chinese adults, we developed 10-year ASCVD risk prediction models that do not require blood testing [20]. The aim of the study was to explore the optimal risk thresholds for initiating statin therapy using the non-laboratory-based soft and hard ASCVD models among the overall population and sub-sets by age, sex, and rural/urban residence separately. We further compared the utility of soft and hard models as screening tools from a cost-utility perspective.

## Methods

### Model overview

**Fig. 1:**
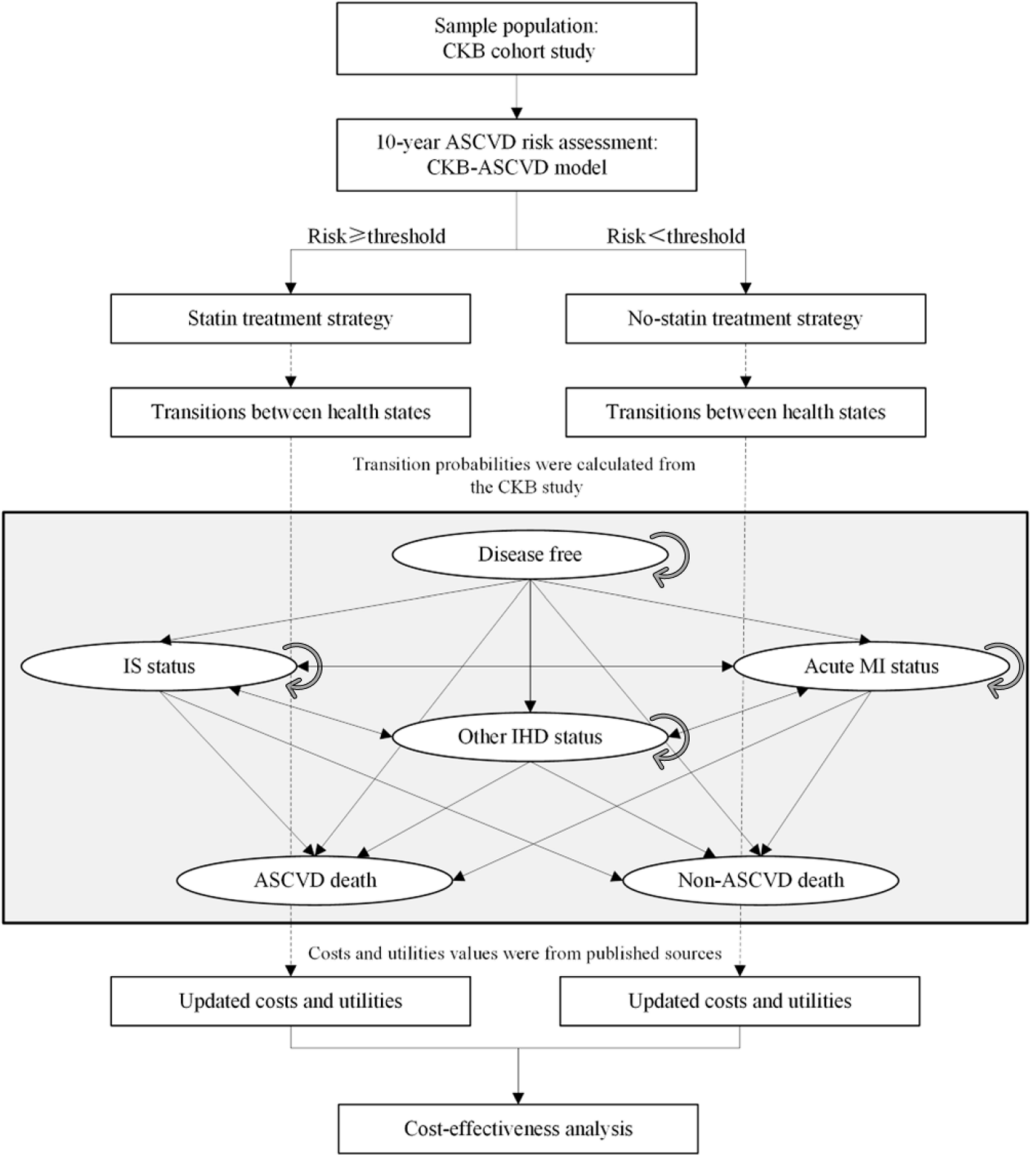
Study overview. CKB, China Kadoorie Biobank; ASCVD, atherosclerotic cardiovascular disease; IS, ischemic stroke; MI, myocardial infarction; IHD, ischemic heart disease

We constructed a decision-analytic Markov cohort model to estimate the cost-utility of different threshold strategies for initiating statin therapy from a societal perspective (Fig. 1). The model projected the incidence of ASCVD events, ASCVD-related costs, and quality-adjusted life years (QALYs) using a lifetime horizon.

According to the natural history of ASCVD, we built this model by referring to previously published CVD Markov models [13, 16, 21]. The health states in the Markov model include disease-free state, non-fatal IHD, non-fatal IS, fatal ASCVD, and fatal non-ASCVD outcomes, respectively. Since there is a significant difference in hospitalization costs between acute MI and other IHD events, we separated these two non-fatal IHD events into two distinct states. The possible transitions between these states are shown in Fig. 1 and Additional file 1: Fig. S1. Individuals without ASCVD at baseline enter the model and will experience the first non-fatal ASCVD event or fatal event. Non-fatal ASCVD events were divided into the acute stage (during the hospitalization) and the chronic stage (after the hospitalization). Considering the possibility of ASCVD comorbidity, patients with the first non-fatal ASCVD event can experience other ASCVD events later. The transitions between non-fatal ASCVD events are bidirectional. Individuals with non-fatal ASCVD events can progress to fatal ASCVD or non-ASCVD events, which are absorbing states.

Each health state in this model was assigned an annual cost and a utility value. All costs were inflated to the 2019 US dollars (1 US$ = 6.9 RMB) [22]. The risk prediction models were used to stratify high- and low-risk population. The transition probabilities were estimated from the CKB study, and all other model parameters were from published sources (Table 1). The model was developed using Treeage Pro 2022 (TreeAge Software; Williamstown, MA, USA).

**Table 1:** Parameters used in the cost-utility model

	Base-case value	Range	Distribution	Source
Statin efficacy and risks				
Relative risk				
Non-fatal IHD events	0.67	0.59–0.76^a^	Log-normal	[28]
Non-fatal IS events	0.69	0.58–0.83^a^	Log-normal	[28]
Fatal ASCVD events	0.83	0.72–0.96^a^	Log-normal	[28]
Discontinuation for adverse events, %				
Diabetes-related events	0.30	0.26–0.35^b^	*β*	[29]
Definite myopathy events	0.24	0.20–0.28^b^	*β*	[29]
Discontinuation for other reasons,%	11.86	10.08–13.64^b^	*β*	[29]
Direct costs, 2019 US$				
Statin related				
Statin costs/year	61.43	40.20–112.07	*γ*	[30]
Examination for ASCVD risk assessment/visit	4.35	2.90–7.25	*γ*	–
Registration fee/year	52.17	34.78–86.96	*γ*	–
Adverse events related				
Diabetes hospitalization/visit	1149.70	977.25–1322.14^b^	*γ*	[31]
Chronic stage of diabetes/year	835.87	710.49–961.25^b^	*γ*	[32]
Definite myopathy events hospitalization/visit	1793.36	1524.35–2062.36^b^	*γ*	[31]
ASCVD related				
Hospitalization/visit				
Acute MI	4401.23	2697.80–4738.36^c^	*γ*	[31]
Other IHD	2037.71	1093.93–2372.42^c^	*γ*	[31]
IS	1421.91	944.87–1830.20^c^	*γ*	[31]
Chronic stage/year				
IHD	427.04	362.99–491.10^b^	*γ*	[33]
IS	242.38	206.01–278.74^b^	*γ*	[33]
Indirect costs				
Duration of hospitalization (day)				
Acute MI	8.05	7.59–8.30^c^	*γ*	[31]
Other IHD	7.79	7.73–7.85^c^	*γ*	[31]
IS	10.01	9.50–10.73^c^	*γ*	[31]
Diabetes-related	9.39	8.83–9.88^c^	*γ*	[31]
Definite myopathy events	9.21	9.03–9.37^c^	*γ*	[31]
Labor related				
Average yearly salaries, 2019 US$	13,116.09	11,148.68–15,083.51^b^	*γ*	[26]
Unemployment rate,%	5.2	4.4–6.0^b^	*β*	[26]
Utility weights				
Disease free				
No statin treatment	1.00		–	
On statin treatment	0.999	0.998–1.000	*β*	[21]
IHD				
Acute stage	0.50	0.20–0.80	–	
Chronic stage	0.87	0.75–0.90	*β*	[37]
IS				
Acute stage	0.50	0.20–0.80	–	
Chronic stage	0.90	–	–	[37]
Adverse events				
Diabetes	0.84	0.79–0.94	*β*	[37]
Myopathy events	0.56	–	–	[37]

*IHD* ischemic heart disease, *IS* ischemic stroke, *ASCVD* atherosclerotic cardiovascular disease, *MI* myocardial infarction

^a^Upper and lower bounds were based on 95% confidence intervals

^b^Upper and lower bounds were based on ±15% of base-case value

^c^Upper and lower bounds were urban and rural values, respectively

### Overview of the CKB study

To simulate the Markov cohort model, we used data from the CKB study. The CKB is a nationwide prospective cohort study, with 512,723 participants aged 30–79 years enrolled from 5 urban and 5 rural areas in China during 2004–2008. Details of the study design, methods, and population characteristics have been reported previously [23]. Briefly, all participants completed an interviewer-administered laptop-based questionnaire, physical measurements, and blood sample collection at baseline by trained health workers after providing written informed consent. This study included 487,570 participants aged 30–75 without ASCVD and statin treatment at baseline. Individuals over 75 years were excluded because of the uncertainty regarding the effects of statins on older adults [24].

Recruited participants in the CKB were followed up through linkages with death and disease registries and health insurance databases and active follow-up. Trained staff blinded to baseline information coded all cases using the 10th revision of the International Classification of Diseases (ICD-10). In this study, the hard ASCVD outcomes were defined as non-fatal MI (I21–I23), fatal IHD (I20–I25), and fatal or non-fatal IS (I63). The soft ASCVD outcomes were defined as all fatal or non-fatal IHD (I20–I25) and IS (I63) events. IHD events were further classified into acute MI (I21) and other IHD events (I20, I22–I25). The loss to follow-up was < 1% until censoring on December 31, 2018.

### Risk assessment

Based on 10-year ASCVD risk prediction models we developed in the CKB cohort (CKB-ASCVD model) [20], we assessed the ASCVD risk for individuals in a disease-free state. The CKB-ASCVD model included age, systolic and diastolic blood pressure, use of blood pressure–lowering treatment, current daily smoking, self-reported history of diabetes, and waist circumference as predictors. The risk prediction model was recalibrated to account for the differences in absolute risk of IHD and IS among different regions of China. We recalibrated the model using observed 10-year risks in each study region, following the recalibration approach proposed by the WHO CVD Risk Chart Working Group [25].

We predicted the 10-year risk of hard and soft ASCVD outcomes separately. The model we have published previously [20] was for predicting soft ASCVD outcomes. Using a similar strategy, we constructed a model to predict hard ASCVD outcomes. The soft and hard ASCVD models had good discrimination and calibration performances in participants aged 30–75. The Harrell *C* for the soft ASCVD model was 0.775 (95% CI: 0.773–0.777) for women and 0.771 (0.769–0.774) for men. The Harrell *C* for the hard ASCVD model was 0.804 (95% CI: 0.802–0.806) for women and 0.794 (95% CI: 0.791–0.797) for men. The calibration performance of the models assessed by comparing the predicted risks with observed risks is shown in Additional file 1: Fig. S2.

### Treatment strategies

Current guidelines on the primary prevention of cardiovascular disease (CVD) recommend using 10-year absolute ASCVD risk to guide decision-making for statin therapy [5–8]. We, therefore, considered the participants with a risk higher than the ASCVD risk threshold eligible for initiating statin therapy. The optimal ASCVD risk threshold for initiating statin therapy was evaluated separately for the hard and soft ASCVD models. We set the risk threshold at a range of 10% to 20% for the soft ASCVD model and 5% to 15% for the hard ASCVD model, with an interval of 1%. The threshold values for the hard ASCVD model were referenced from established guidelines and previous studies, while those for the soft ASCVD model were selected to ensure comparability in the proportion of high-risk population with the hard ASCVD model. We also included a threshold of 7.5% for the hard ASCVD model recommended by the ACC/AHA [5] and the no treatment strategy.

### Model parameters

#### Transition probabilities

The transition probabilities between different health states were derived from CKB data. We calculated the 10-year age-sex-specific ASCVD morbidity and mortality and non-ASCVD mortality (in 5-year age groups, including 30–34, 35–39, 40–44, 45–49, 50–54, 55–59, 60–64, 65–69, and 70–75) in the CKB population. The probability was weighted according to the national population sampling survey data in 2019 by sex and age groups [26]. Ten-year transition probability was then converted to 1-year transition probability using the formula *r* = 1 − exp(ln(1 − *p*)/10), where *r* denotes the 1-year incidence and *p* represents the cumulative incidence over 10 years [27]. Since the transition probability from the disease-free state to the first ASCVD event is greatly affected by the baseline risk level, we calculated weighted 10-year morbidity or mortality for populations eligible and non-eligible for statins separately by different ASCVD risk thresholds. When comparing strategy with the highest threshold (20% for soft and 15% for hard ASCVD models) with no treatment strategy, we uniformly used transition probabilities of the former to make them comparable. The proportion of individuals eligible for statins was also weighted by sex and age distribution in the Chinese population.

Since the number of incident cases from the first ASCVD event to other states was low in the CKB population, we used the average morbidity or mortality of the whole population as the transition probability. The transition probabilities used in the model are shown in Additional file 1: Table S1.

#### Statin treatment

The efficacy of statins on non-fatal IHD events, non-fatal IS events, and fatal ASCVD events was derived from a 2013 meta-analysis of 18 randomized trials [28]. The incidence of side effects caused by statins and statin compliance rates were derived from the data of the 5468 Chinese participants in the HPS2-THREVE randomized controlled trial who used simvastatin only [29]. This trial indicated a higher risk of myopathy events associated with statins in the Chinese than in the European populations.

We simulated diabetes and myopathy as adverse events in the model, assuming that individuals experiencing adverse events will no longer receive drug interventions (Additional file 1: Fig. S1). Myopathy was defined as unexplained muscle symptoms with a creatine kinase > 10 × upper limit of normal in HPS2-THREVE study [29]. In addition, the model also simulated discontinuation for other reasons. Since undiagnosed myopathy, liver disease, and other adverse events have little impact on patients and can be self-treated through drug withdrawal, they were only included in the statin discontinuation, and their costs and disutilities were not considered in the model of this study.

#### Costs and utilities

From a societal perspective, we included direct and indirect costs in this study. For direct costs, we considered the costs associated with statin therapy and ASCVD treatment, including annual statin costs, annual registration fee, examination for ASCVD risk assessment, hospitalization costs of adverse events, hospitalization costs of ASCVD, and costs of ASCVD treatment in the chronic stage.

The prices of statins were obtained from the centralized procurement policy bid-winning announcement file [30]. Considering that high-intensity statin treatment in the Chinese population has relatively high side effects, the daily dose (pitavastatin 4 mg/day) was calculated with moderate intensity according to the Chinese guidelines [8]. Since the predictors in the CKB-ASCVD model did not include invasive tests such as blood lipids, the risk assessment fee corresponds to the registration fee for a hospital visit. Hospitalization costs were from the China Health Statistical Yearbook in 2020 [31]. The costs of ASCVD treatment in the chronic stage were derived from the published studies of the Chinese population [32, 33].

Indirect costs, referring to productivity loss due to disease, disability, or death, were calculated by the human capital approach [34]. We used the national average salary from the labor market in 2019 to estimate the productivity loss caused by hospitalization or premature death for patients and caregiving for one family member during hospitalization, assuming all lost time will be used for production [26]. Relevant data were from the China Health Statistics Yearbook and China Statistical Yearbook in 2020 [26, 31].

The utility values of each health state were estimated to calculate QALYs. The utility of a disease-free state without statin treatment was set as 1, and the utility of death was 0. Referring to previous studies, we set the utility of statin treatment as 0.999 [21], and the utility of acute ASCVD events as 0.5 [35, 36]. The time spent on acute events was considered as the duration of hospitalization. Utility values for the remaining states were obtained from published surveys of the Chinese population [37].

### Statistical analyses

We calculated incremental cost-effectiveness ratios (ICERs) between the no treatment and the different threshold strategies to explore the optimal thresholds for the soft and hard ASCVD models separately. ICER values were estimated by comparing adjacent strategies sequentially according to the thresholds from low to high. Strategies were ruled out through extended dominance (larger ICER than a more effective strategy) or dominated (higher costs and lower QALYs) [38]. We also compared the cost-utility of the soft and hard ASCVD models by setting approximately the same proportion of the treated population referring to a previous study [39].

We used the WTP of the Chinese GDP per capita in 2019 ($10,274) to determine the optimal ASCVD risk threshold strategies as recommended by the Commission on Macroeconomics and Health [40]. We also used three times the GDP per capita as an upper WTP ($30,823). As WHO recommended, the intervention is not cost-effective if it costs more than three times the GDP per capita [41]. Both costs and utility values were discounted at 5% per year in the base-case analysis and ranged from 0 to 8% in the sensitivity analysis according to the China pharmacoeconomic guidelines [42].

We performed one-way sensitivity analyses to assess the variability resulting from the changes in main parameters. When 95% confidence intervals or other values were unavailable from the source data, ± 15% of a base-case value was used to generate upper and lower bounds. We performed probabilistic sensitivity analyses (PSA) using Monte Carlo simulations (*N* = 1000 iterations) to assess the joint uncertainty of the parameters that populated the Markov model. The lower and upper bounds used in the one-way sensitivity analyses and the distribution of parameters used in the probabilistic sensitivity analyses are listed in Table 1.

Stratified analyses were performed by age (30–59 years and 60–75 years), region (urban and rural areas), and sex. Transition probabilities of these subgroups for the incremental analysis were calculated separately using CKB data. Probability was weighted by age group in sex-specific analyses, by sex in age-stratified analyses, and by both age group and sex in region-specific analyses. In the stratification analysis by region, the parameters of ASCVD hospitalization costs and length of stay were the respective values of the urban and rural populations from the China Health Statistical Yearbook in 2020 [31]. The reporting of methods and results conformed to the Consolidated Health Economic Evaluation Reporting Standards (Additional file 1: Supplemental Method) [43].

## Results

### Study population

Of 487,571 participants, 82,575 (16.9%) experienced soft ASCVD outcomes, and 55,704 (11.4%) experienced hard ASCVD outcomes during a median of 12 years of follow-up (Additional file 1: Table S2). The proportion of women with soft ASCVD outcomes was higher than that with hard ASCVD outcomes.

### Model validation

To examine the internal validity of the model, we calculated the observed probabilities of all fatal and non-fatal events in the CKB population over 10 years. The simulated probabilities were slightly higher than the observed probabilities in the CKB population (Additional file 1: Fig. S3). In external validation, we compared the simulated all-cause mortality with the mortality data of the Chinese population from the Global Burden of Disease (GBD) study 2019 [3]. The simulated mortality was comparable with the mortality from GBD mostly (Additional file 1: Fig. S4).

### Optimal threshold strategies

In the base-case analyses, using a WTP of the GDP per capita in 2019, the optimal ASCVD risk threshold was 18% (ICER compared with the 19% threshold, $7013.48/QALY) for the soft ASCVD model and 10% (ICER compared with the 11% threshold, $6540.71/QALY) for the hard ASCVD model, respectively (Table 2). The proportion of women in the high-risk population defined by the 18% threshold for soft ASCVD model is higher than that defined by the 10% threshold for hard ASCVD model (Additional file 1: Table S3). The threshold of 7.5% for the hard ASCVD model was not cost-effective under the WTP of three times the GDP per capita (ICER compared with the 8% threshold, $122,746.23/QALY) (Table 2).

**Table 2:** Cost-utility of ASCVD threshold strategies for statin treatment

ASCVD risk threshold (%)	Statin eligible (%)	ASCVD events (%)^a^	Costs (2019 US$)	QALYs	ICER (US$/QALY)
Soft ASCVD model					
No treatment	0	17.22	2896.97	13.9445	Ref
20	25.4	15.49	3058.69	13.9995	2938.39
19	26.9	15.38	3069.51	14.0020	4285.01
18	28.5	15.29	3082.78	14.0039	7013.48
17	30.2	15.21	3099.47	14.0051	13,776.82
16	32.0	15.15	3118.27	14.0059	Extended dominance^b^
15	33.9	15.08	3137.52	14.0070	19,941.10
14	36.0	15.00	3160.65	14.0078	31,057.34
13	38.3	14.94	3186.38	14.0083	47,434.92
12	40.8	14.89	3216.52	14.0080	Dominated^c^
11	43.6	14.87	3252.31	14.0068	Dominated^c^
10	46.6	14.85	3292.01	14.0054	Dominated^c^
Hard ASCVD model					
No treatment	0	17.42	2904.86	13.9412	Ref
15	19.6	16.10	3031.48	13.9846	Extended dominance^b^
14	21.3	15.92	3038.66	13.9893	1532.69
13	23.1	15.77	3049.30	13.9932	2781.73
12	25.1	15.61	3061.80	13.9974	2979.34
11	27.3	15.46	3078.87	14.0007	5127.38
10	29.7	15.32	3099.32	14.0038	6540.71
9	32.6	15.19	3126.75	14.0058	13,516.35
8	35.7	15.08	3160.21	14.0071	27,364.86
7.5	37.5	15.04	3180.38	14.0072	122,746.23
7	39.4	15.00	3203.11	14.0071	Dominated^c^
6	43.6	14.92	3255.13	14.0065	Dominated^c^
5	48.6	14.91	3322.82	14.0034	Dominated^c^

*ASCVD* atherosclerotic cardiovascular disease, *QALYs* quality-adjusted life year, *ICER* incremental cost-effectiveness ratio

^a^Proportion of individuals who will experience ASCVD events in their lifetime

^b^Extended dominance indicates larger ICER than a more effective strategy

^c^Dominated indicates higher cost and lower QALYs than the comparator

The thresholds of optimal strategies were more sensitive to variations in statin-related costs, the effects of statin, and discount rate in the one-way sensitivity analyses (Additional file 1: Figs. S5 and S6). However, the above optimal threshold strategies were relatively robust to the range of parameters. Among all the parameters, statin price had the greatest effect on cost-utility estimates. Specifically, the ICERs would be slightly beyond the GDP per capita for a soft ASCVD model threshold of 18% and a hard ASCVD model threshold of 10% when using the upper bound of the annual statin costs (ICER $11,248.25/QALY and $10,913.03/QALY).

**Fig. 2:**
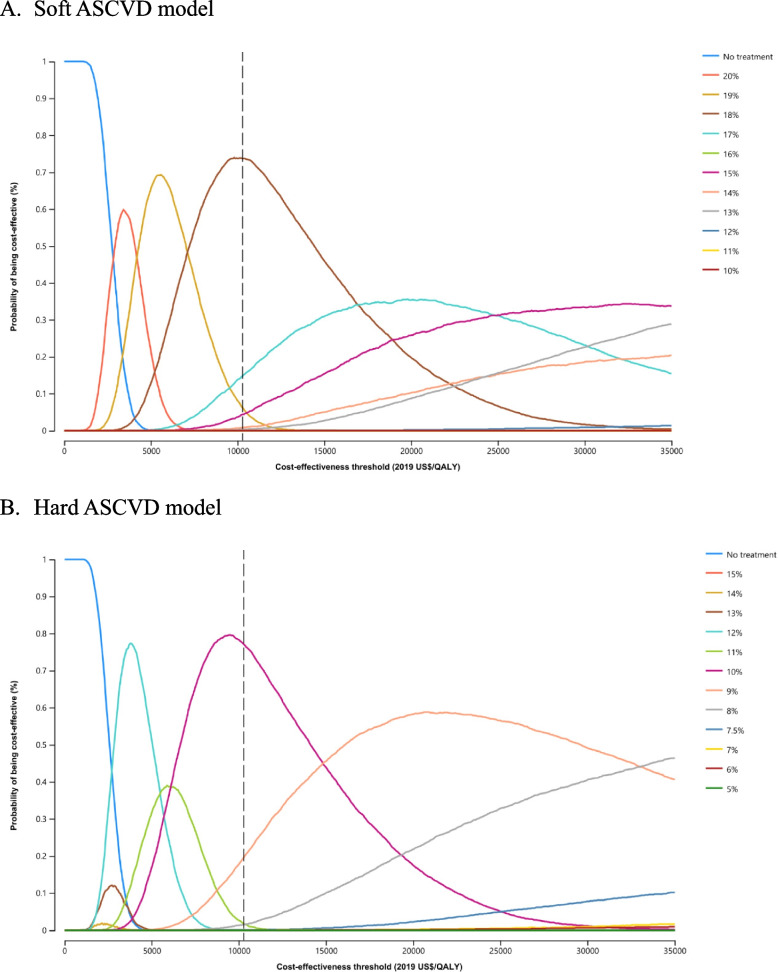
Cost-utility acceptability curves for ASCVD threshold strategies. ASCVD, atherosclerotic cardiovascular disease; QALYs, quality-adjusted life year. Dashed lines indicate the GDP per capita in 2019

The acceptability curves in Fig. 2 show the PSA results. Using the WTP of the GDP per capita, the soft ASCVD model threshold of 18% was optimal in 73% of PSA iterations. Similarly, more than 77% of PSA iterations had an optimal threshold of 10% for the hard ASCVD model.

### Optimal threshold strategies in subpopulations

Given the same ASCVD risk thresholds, the proportions of statin eligibility were significantly higher in the 60–75 age group than in the 30–59 age groups (Table 3 and Additional file 1: Table S4). The soft and hard ASCVD model thresholds of 18% and 10% were cost-effective in the 30–59 age group, and the optimal thresholds could reduce to 13% and 9%, respectively (Additional file 1: Figs. S7–S10). In contrast, the 18% and 10% thresholds were not cost-effective in the 60–75 age group, with ICERs beyond three times the GDP per capita ($39,293.81/QALY and $38,453.64/QALY, respectively). The optimal thresholds for this age group were 20% and 15%, respectively (Additional file 1: Figs. S11–S14).

**Table 3:** Cost-utility of ASCVD threshold strategies for soft ASCVD model by age

ASCVD risk threshold (%)	30–59 years				60–75 years			
	Statin eligible (%)	Costs (2019 US$)	QALYs	ICER (US$/QALY)	Statin eligible (%)	Costs (2019 US$)	QALYs	ICER (US$/QALY)
No treatment	0	2631.12	15.3115	Ref	0	594.47	7.7303	Ref
20	10.4	2738.66	15.3309	Extended dominance^a^	76.0	1027.76	7.7842	8033.19
19	11.6	2744.67	15.3334	2376.27	78.5	1045.31	7.7848	32,512.82
18	12.9	2753.40	15.3357	5052.67	81.1	1062.60	7.7852	39,293.81
17	14.4	2766.06	15.3376	6454.37	83.4	1079.46	7.7855	54,177.84
16	16.1	2780.29	15.3398	Extended dominance^a^	85.6	1095.50	7.7856	Extended dominance^a^
15	18.0	2796.22	15.3423	6469.71	87.5	1109.24	7.7858	121,207.51
14	20.2	2816.21	15.3449	7589.41	89.4	1122.76	7.7858	347,687.13
13	22.7	2839.49	15.3476	8823.45	91.1	1135.70	7.7858	862,135.82
12	25.5	2868.64	15.3497	13,688.91	92.7	1147.44	7.7858	Dominated^b^
11	28.6	2904.53	15.3515	20,452.37	94.1	1158.44	7.7856	Dominated^b^
10	32.1	2946.04	15.3531	25,720.71	95.5	1168.64	7.7855	Dominated^b^

*ASCVD* atherosclerotic cardiovascular disease, *QALYs* quality-adjusted life year, *ICER* incremental cost-effectiveness ratio

^a^Extended dominance indicates larger ICER than a more effective strategy

^b^Dominated indicates higher cost and lower QALYs than the comparator

The optimal threshold of 18% for the soft ASCVD model and 10% for the hard ASCVD model were mostly robust in stratified analyses by region and sex (Additional file 1: Tables S5–S8). The ICER would be slightly beyond the GDP per capita for the hard ASCVD model threshold of 10% in males (ICER compared with the 11% threshold, $11,472.03/QALY). The threshold of 7.5% for the hard ASCVD model was cost-effective at three times the GDP per capita for females (ICER compared with the 8% threshold, $17,068.80/QALY).

### Comparison of soft and hard ASCVD models

For each individual, the 10-year ASCVD risk predicted by the soft ASCVD model was higher than that by the hard ASCVD model. The median predicted 10-year ASCVD risk was 9.8% for soft outcomes and 5.2% for hard outcomes in the CKB population. Using the same ASCVD risk threshold, the proportion eligible for statins was higher for the soft than hard ASCVD models (Table 2).

When setting approximately the same proportion of high-risk individuals receiving statin therapy, the strategy of risk assessment and statin initiation based on the soft ASCVD model was mostly dominant compared to that based on the hard ASCVD model (Table 4). More importantly, when using the threshold of 18% for the soft ASCVD model and 10% for the hard ASCVD model, the optimal thresholds at the GDP per capita, the strategy based on the soft model would experience fewer ASCVD outcomes (15.29% vs. 15.32%), cost less ($3082.78 vs. $3099.32), and have higher QALYs (14.0039 vs.14.0038) than that based on the hard model.

**Table 4:** Comparative cost-utility of ASCVD threshold strategies between hard and soft ASCVD models^a^

ASCVD risk threshold (%)	Statin eligible (%)	ASCVD events (%)^b^	Costs (2019 US$)	QALYs	ICER (US$/QALY)
12	25.1	15.61	3061.80	13.9974	Ref
20	25.4	15.49	3058.69	13.9995	Strongly dominant^c^
11	27.3	15.46	3078.87	14.0007	Ref
19	26.9	15.38	3069.51	14.0020	Strongly dominant^c^
10	29.7	15.32	3099.32	14.0038	Ref
17	30.2	15.21	3099.47	14.0051	115.38
8	35.7	15.08	3160.21	14.0071	Ref
14	36.0	15.00	3160.65	14.0078	73.23
6	43.6	14.92	3255.13	14.0065	Ref
11	43.6	14.87	3252.31	14.0068	Strongly dominant^c^

*ASCVD* atherosclerotic cardiovascular disease, *QALYs* quality-adjusted life year, *ICER* incremental cost-effectiveness ratio

^a^Grey area indicates the threshold strategies of soft model. White area indicates the threshold strategies of hard model

^b^Proportion of individuals who will experience ASCVD events in their lifetime

^c^Strongly dominant indicates lower cost and higher QALYs than the comparator

## Discussion

Based on non-laboratory-based ASCVD risk prediction models, we identified that using thresholds of 18% for the soft ASCVD model and 10% for the hard model for statin initiation would be cost-effective for individuals aged 30–75 years, costing less than the GDP per capita in China per QALY gained. These optimal thresholds were robust in most sensitivity and stratified analyses by region and sex. Based on age-stratified findings, we recommend implementing higher threshold values for older populations. From the cost-utility perspective, using the soft ASCVD model as the screening tool was strongly dominant compared to the hard ASCVD model.

Only one study in China has explored the optimal threshold for a laboratory-based risk prediction model developed by Prediction for ASCVD Risk in China (China-PAR) [17]. This study estimated the distributions of ASCVD risk factors using data from the China Health and Retirement Longitudinal Study (CHARLS) of participants aged 45 to 89 years and used the 10-year risk of hard ASCVD outcomes predicted by the China-PAR model as the transition probabilities of ASCVD. The results showed that the risk threshold of 10% was cost-effective compared with the threshold of 15% only under the WTP of three times the per capita GDP (ICER $22,455.65/QALY). In our study, the ICER for the hard ASCVD model threshold of 10% was $3511.46/QALY ([$3099.32–$3031.48])/[14.0038–13.9846]) compared with the threshold of 15%, costing less than the GDP per capita per QALY gained. The discrepancy in the results may be because the previous study did not consider the occurrence of non-fatal IHD events, which are very common among the Chinese population [19], and underestimated the effectiveness of statin therapy. Also, the previous study did not consider that statin therapy would reduce productivity loss due to illness or early death, therefore may overestimate the ICER. In contrast, our study used data from the CKB cohort to estimate transition probabilities for all possible pathways involving fatal and non-fatal events, and considered direct and indirect costs of ASCVD.

The present study showed that statin prices had the greatest impact on cost-utility estimates. A previous study found that statins for primary prevention of ASCVD were cost-effective only after the implementation of China’s centralized medicine procurement policy in 2019, which led to a significant decline in statin prices [16]. This study population aged 35 to 64 years was derived from the Chinese Multi-provincial Cohort Study (CMCS), and the 10-year risk of hard ASCVD outcomes predicted by the CMCS model was used as the probabilities of ASCVD. High-risk individuals eligible for statins were defined by dyslipidemia and a 10-year ASCVD risk threshold of 10%. Compared with no treatment strategy, the ICER was below two times the GDP per capita per QALY gained. The corresponding ICER for the hard ASCVD model threshold of 10% in our study was $3106.39/QALY ([$3099.32–$2904.86]/[14.0038–13.9412]). Reasons for a higher ICER in the previous study may be due to a 10-year horizon used, the failure to account for the occurrence of non-fatal IHD events, and higher statin treatment-related costs (i.e., registration fee and costs of lipid testing for risk assessment).

The 10-year ASCVD risk threshold of 7.5% used in the ACC/AHA guidelines was proven to be cost-effective in US adults aged 45 to 75 years, and thresholds of 4.0% or 3.0% would be considered optimal using more lenient WTP standards [13]. However, such a low threshold was not cost-effective in our population. The higher benefits of statin therapy in US adults may be due to the lower cost of statin use [36] and the higher cost of ASCVD treatment. The comparison suggests that it is necessary to conduct cost-utility analyses separately in populations with different socioeconomic and healthcare resources to determine the appropriate risk threshold for statin initiation.

Most current guidelines recommend the same threshold for statin treatment for all age groups, except that the 2021 European Society of Cardiology (ESC) guidelines recommend different thresholds for different age groups (i.e., thresholds of 7.5%, 10%, and 15% for adults aged < 50, 50–69, and > 70 years, respectively) [6]. Our findings also suggested higher optimal thresholds for older than younger adults from a cost-utility perspective. At lower thresholds, treatment rates in the older population were too high, leading to potential overtreatment, and therefore statin treatment may not be cost-effective. In contrast, a previous cost-utility study conducted in the Netherlands found that statin treatment was more cost-effective in the elderly [21]. The opposite result could be attributed to the healthcare payer’s perspective used in this study, which only included direct medical costs. The reduction in indirect costs due to statin treatment is mainly from the middle-aged labor force, and ignoring this part could underestimate the cost-utility among younger adults.

The risk thresholds for statin initiation differ depending on the outcome definition of the ASCVD risk prediction model [44]. The EPIC-NL cohort study followed 15,880 participants aged 35 to 65 for 10 years and found that the absolute risks of hard cardiovascular outcomes were approximately fourfold greater than for CVD mortality [45]. Based on this study, the European guidelines suggested that a SCORE risk of fatal CVD of 5% translates into a hard CVD outcome risk of 15% for men and about 20% for women [46]. In the present study, the predicted 10-year risk of soft ASCVD outcomes was approximately double that of hard ASCVD outcomes (9.8% vs. 5.2%). Correspondingly, the optimal risk threshold for the soft ASCVD model was 18%, about twice the threshold for the hard model (10%).

Current guidelines on CVD primary prevention mostly recommend the use of CVD risk prediction tools that predict the risk of hard outcomes. However, the disability and financial burden caused by non-fatal IHD events should not be ignored [47]. From the cost-utility perspective, as shown in the present study, the use of soft ASCVD model as a screening tool identifies a similar proportion of the population eligible for statin therapy but is strongly dominant compared with the use of hard ASCVD model. To our knowledge, this is the first study to compare the cost and the effectiveness of soft and hard ASCVD models. Further studies are required to confirm these findings in independent populations.

The CKB cohort data used in this study achieved long-term follow-up for fatal and non-fatal IHD and IS events by linking to health insurance records, disease, and death registers. The estimation of transition probabilities was based on actual observations in the CKB population rather than on published studies of different populations to ensure the homogeneity of transition probabilities. The study has several limitations. First, because of the lack of evidence on the efficacy of statin therapy and the utility of statin underuse and acute ASCVD events in the Chinese population, these parameters were obtained from trials in other countries or from previous studies. However, sensitivity analyses indicated that the baseline results were robust over a wide range of parameter inputs. Second, we used the 10-year average morbidity and mortality as the transition probabilities from the disease-free state to ASCVD, which would underestimate the lifetime ASCVD risk and the benefit of statin treatment, resulting in conservative estimates. Third, we assumed lifelong statin treatment and 100% efficacy over the lifetime, consistent with previous studies [13, 48]. However, our one-way sensitivity analyses showed that the results were robust to the rate of discontinuation and the efficacy of statin treatment. Also, a previous study indicated that the cost-utility of statin treatment was less affected by the duration of statin treatment [17]. Fourth, although individuals with statin use at baseline were excluded from calculating transition probabilities, we could not identify individuals initiating statin therapy during follow-up in the remaining population. Finally, despite recalibration, ASCVD prediction models for baseline risk stratification retained a potential for misclassification.

## Conclusions

In this cost-utility analysis of Chinese adults aged 30 to 75 years, the risk threshold of 18% for the soft ASCVD model and 10% for the hard ASCVD model have acceptable cost-utility profiles. Using different thresholds for statin treatment based on age is an alternative strategy that requires further evaluation. In addition, the soft ASCVD model, considering all fatal and non-fatal events, was more cost-effective than the hard ASCVD model and should be used as a screening tool for CVD primary prevention.

## Supplementary Information

Additional file 1: Supplemental Method—CHEERS 2022 Checklist. Figures S1–S14. Fig. S1 Structure of the Markov model; Fig. S2 Calibration plots for CKB hard and soft ASCVD models after recalibration; Fig. S3 Observed vs. simulated probabilities of all events over 10 years; Fig. S4 GBD vs. simulated age-sex-specific all-cause mortality; Fig. S5 One-way sensitivity analyses for soft ASCVD model threshold of 18% vs. 19%; Fig. S6 One-way sensitivity analyses for hard ASCVD model threshold of 10% vs. 11%; Fig. S7 One-way sensitivity analyses for soft ASCVD model threshold of 13% vs. 14% in population aged 30–59 years; Fig. S8 Cost-utility acceptability curves for soft ASCVD threshold strategies in population aged 30–59 years; Fig. S9 One-way sensitivity analyses for hard ASCVD model threshold of 9% vs. 10% in population aged 30–59 years; Fig. S10 Cost-utility acceptability curves for hard ASCVD threshold strategies in population aged 30–59 years; Fig. S11 One-way sensitivity analyses for soft ASCVD model threshold of 20% vs. no treatment in population aged 60–75 years; Fig. S12 Cost-utility acceptability curves for soft ASCVD threshold strategies in population aged 60–75 years; Fig. S13 One-way sensitivity analyses for hard ASCVD model threshold of 15% vs. no treatment in population aged 60–75 years; Fig. S14 Cost-utility acceptability curves for hard ASCVD threshold strategies in population aged 60–75 years. Tables S1–S8. Table S1 Disease progression inputs used in the cost-utility model; Table S2 Baseline characteristics of participants with soft or hard ASCVD outcomes occurred during follow-up in CKB study; Table S3 Baseline characteristics of high-risk population defined by optimal thresholds for soft and hard ASCVD models; Table S4 Cost-utility of ASCVD threshold strategies for hard ASCVD model by age; Table S5 Cost-utility of ASCVD threshold strategies for soft ASCVD model by region; Table S6 Cost-utility of ASCVD threshold strategies for hard ASCVD model by region; Table S7 Cost-utility of ASCVD threshold strategies for soft ASCVD model by sex; Table S8 Cost-utility of ASCVD threshold strategies for hard ASCVD model by sex.

## Data Availability

The access policy and procedures are available at www.ckbiobank.org.

## Abbreviations

ASCVD: Atherosclerotic cardiovascular disease
IHD: Ischemic heart disease
IS: Ischemic stroke
ACC/AHA: American College of Cardiology and American Heart Association
WTP: Willingness to pay
GDP: Gross domestic product
MI: Myocardial infarction
CKB: China Kadoorie Biobank
QALYs: Quality-adjusted life years
ICD-10: International Classification of Diseases, Tenth Revision
CVD: Cardiovascular disease
ICER: Incremental cost-effectiveness ratio
PSA: Probabilistic sensitivity analyses
GBD: Global Burden of Disease
China-PAR: Prediction for ASCVD Risk in China
CHARLS: China Health and Retirement Longitudinal Study
