# Supplementary material for: Risk thresholds for soft versus hard cardiovascular disease outcome models for initiating statin therapy among Chinese adults: a cost-utility analysis
Source: BMC Med. 2025 Jul 1;23:353. doi: 10.1186/s12916-025-04222-8 (PMC12211359; doi:10.1186/s12916-025-04222-8)
Supplement: Supplementary file 1 — Additional file 1: Supplemental Method—CHEERS 2022 Checklist. Figures S1–S14. Fig. S1 Structure of the Markov model; Fig. S2 Calibration plots for CKB hard and soft ASCVD models after recalibration; Fig. S3 Observed vs. simulated probabilities of all events over 10 years; Fig. S4 GBD vs. simulated age-sex-specific all-cause mortality; Fig. S5 One-way sensitivity analyses for soft ASCVD model threshold of 18% vs. 19%; Fig. S6 One-way sensitivity analyses for hard ASCVD model threshold of 10% vs. 11%; Fig. S7 One-way sensitivity analyses for soft ASCVD model threshold of 13% vs. 14% in population aged 30–59 years; Fig. S8 Cost-utility acceptability curves for soft ASCVD threshold strategies in population aged 30–59 years; Fig. S9 One-way sensitivity analyses for hard ASCVD model threshold of 9% vs. 10% in population aged 30–59 years; Fig. S10 Cost-utility acceptability curves for hard ASCVD threshold strategies in population aged 30–59 years; Fig. S11 One-way sensitivity analyses for soft ASCVD model threshold of 20% vs. no treatment in population aged 60–75 years; Fig. S12 Cost-utility acceptability curves for soft ASCVD threshold strategies in population aged 60–75 years; Fig. S13 One-way sensitivity analyses for hard ASCVD model threshold of 15% vs. no treatment in population aged 60–75 years; Fig. S14 Cost-utility acceptability curves for hard ASCVD threshold strategies in population aged 60–75 years. Tables S1–S8. Table S1 Disease progression inputs used in the cost-utility model; Table S2 Baseline characteristics of participants with soft or hard ASCVD outcomes occurred during follow-up in CKB study; Table S3 Baseline characteristics of high-risk population defined by optimal thresholds for soft and hard ASCVD models; Table S4 Cost-utility of ASCVD threshold strategies for hard ASCVD model by age; Table S5 Cost-utility of ASCVD threshold strategies for soft ASCVD model by region; Table S6 Cost-utility of ASCVD threshold strategies for hard ASCVD model by region; [file 12916_2025_4222_MOESM1_ESM.docx]

**Risk thresholds for soft versus hard cardiovascular disease outcome models for initiating statin therapy among Chinese adults: a cost-utility analysis**

**Supplementary Online Content**

[**Members of the China Kadoorie Biobank collaborative group** 2](#_Toc200449061)

[Supplemental Method 3](#_Toc200449062)

[CHEERS 2022 Checklist 3](#_Toc200449063)

[Supplemental Figures 6](#_Toc200449064)

[Figure S1. Structure of the Markov model 7](#_Toc200449065)

[Figure S2. Calibration plots for CKB hard and soft ASCVD models after recalibration. 8](#_Toc200449066)

[Figure S3. Observed vs. simulated probabilities of all events over 10 years 9](#_Toc200449067)

[Figure S4. GBD vs. simulated age-sex-specific all-cause mortality (1/100000) 10](#_Toc200449068)

[Figure S5. One-way sensitivity analyses for soft ASCVD model threshold of 18% vs. 19% 11](#_Toc200449069)

[Figure S6. One-way sensitivity analyses for hard ASCVD model threshold of 10% vs. 11% 12](#_Toc200449070)

[Figure S7. One-way sensitivity analyses for soft ASCVD model threshold of 13% vs. 14% in population aged 30-59 years 13](#_Toc200449071)

[Figure S8. Cost-utility acceptability curves for soft ASCVD threshold strategies in population aged 30-59 years 14](#_Toc200449072)

[Figure S9. One-way sensitivity analyses for hard ASCVD model threshold of 9% vs. 10% in population aged 30-59 years 15](#_Toc200449073)

[Figure S10. Cost-utility acceptability curves for hard ASCVD threshold strategies in population aged 30-59 years 16](#_Toc200449074)

[Figure S11. One-way sensitivity analyses for soft ASCVD model threshold of 20% vs. no treatment in population aged 60-75 years 17](#_Toc200449075)

[Figure S12. Cost-utility acceptability curves for soft ASCVD threshold strategies in population aged 60-75 years 18](#_Toc200449076)

[Figure S13. One-way sensitivity analyses for hard ASCVD model threshold of 15% vs. no treatment in population aged 60-75 years 19](#_Toc200449077)

[Figure S14. Cost-utility acceptability curves for hard ASCVD threshold strategies in population aged 60-75 years 20](#_Toc200449078)

[Supplemental Tables 21](#_Toc200449079)

[Table S1. Disease progression inputs used in the cost-utility model 21](#_Toc200449080)

[Table S2. Baseline characteristics of participants with soft or hard ASCVD outcomes occurred during follow-up in CKB study 22](#_Toc200449081)

[**Table S3. Baseline characteristics of high-risk population defined by optimal thresholds for soft and hard ASCVD models** 23](#_Toc200449082)

[Table S4. Cost-utility of ASCVD threshold strategies for hard ASCVD model by age 24](#_Toc200449083)

[Table S5. Cost-utility of ASCVD threshold strategies for soft ASCVD model by region 25](#_Toc200449084)

[Table S6. Cost-utility of ASCVD threshold strategies for hard ASCVD model by region 26](#_Toc200449085)

[Table S7. Cost-utility of ASCVD threshold strategies for soft ASCVD model by sex 27](#_Toc200449086)

[Table S8. Cost-utility of ASCVD threshold strategies for hard ASCVD model by sex 28](#_Toc200449087)

**Members of the China Kadoorie Biobank collaborative group**

**International Steering Committee:** Junshi Chen, Zhengming Chen (PI), Robert Clarke, Rory Collins, Yu Guo, Liming Li (PI), Jun Lv, Richard Peto, Robin Walters. **International Co-ordinating Centre, Oxford:** Daniel Avery, Derrick Bennett, Ruth Boxall, Sue Burgess, Ka Hung Chan, Yumei Chang, Yiping Chen, Zhengming Chen, Johnathan Clarke; Robert Clarke, Huaidong Du, Ahmed Edris Mohamed, Zammy Fairhurst-Hunter, Hannah Fry, Simon Gilbert, Alex Hacker, Mike Hill, Michael Holmes, Pek Kei Im, Andri Iona, Maria Kakkoura, Christiana Kartsonaki, Rene Kerosi, Kuang Lin, Mohsen Mazidi, Iona Millwood, Sam Morris, Qunhua Nie, Alfred Pozarickij, Paul Ryder, Saredo Said, Sam Sansome, Dan Schmidt, Paul Sherliker, Rajani Sohoni, Becky Stevens, Iain Turnbull, Robin Walters, Lin Wang, Neil Wright, Ling Yang, Xiaoming Yang, Pang Yao.

**National Co-ordinating Centre, Beijing:** Yu Guo, Xiao Han, Can Hou, Jun Lv, Pei Pei, Chao Liu, Canqing Yu, Qingmei Xia. **10 Regional Co-ordinating Centres: Qingdao CDC:** Zengchang Pang, Ruqin Gao, Shanpeng Li, Haiping Duan, Shaojie Wang, Yongmei Liu, Ranran Du, Yajing Zang, Liang Cheng, Xiaocao Tian, Hua Zhang, Yaoming Zhai, Feng Ning, Xiaohui Sun, Feifei Li. **Licang CDC:** Silu Lv, Junzheng Wang, Wei Hou. **Heilongjiang Provincial CDC:** Wei Sun, Shichun Yan, Xiaoming Cui. **Nangang CDC:** Chi Wang, Zhenyuan Wu,Yanjie Li, Quan Kang. **Hainan Provincial CDC:** Huiming Luo, Tingting Ou. **Meilan CDC:** Xiangyang Zheng, Zhendong Guo, Shukuan Wu, Yilei Li, Huimei Li. **Jiangsu Provincial CDC:** Ming Wu, Yonglin Zhou, Jinyi Zhou, Ran Tao, Jie Yang, Jian Su. **Suzhou CDC:** Fang Liu, Jun Zhang, Yihe Hu, Yan Lu, Liangcai Ma, Aiyu Tang, Shuo Zhang, Jianrong Jin, Jingchao Liu. **Guangxi Provincial CDC:** Mei Lin, Zhenzhen Lu. **Liuzhou CDC:** Lifang Zhou, Changping Xie, Jian Lan,Tingping Zhu,Yun Liu, Liuping Wei, Liyuan Zhou, Ningyu Chen, Yulu Qin, Sisi Wang. **Sichuan Provincial CDC:** Xianping Wu, Ningmei Zhang, Xiaofang Chen, Xiaoyu Chang. **Pengzhou CDC:** Mingqiang Yuan, Xia Wu, Xiaofang Chen, Wei Jiang, Jiaqiu Liu, Qiang Sun. **Gansu Provincial CDC:** Faqing Chen, Xiaolan Ren, Caixia Dong. **Maiji CDC:** Hui Zhang, Enke Mao, Xiaoping Wang, Tao Wang, Xi zhang. **Henan Provincial CDC:** Kai Kang, Shixian Feng, Huizi Tian, Lei Fan. **Huixian CDC:** XiaoLin Li, Huarong Sun, Pan He, Xukui Zhang. **Zhejiang Provincial CDC:** Min Yu, Ruying Hu, Hao Wang. **Tongxiang CDC**: Xiaoyi Zhang, Yuan Cao, Kaixu Xie, Lingli Chen, Dun Shen. **Hunan Provincial CDC:** Xiaojun Li, Donghui Jin, Li Yin, Huilin Liu, Zhongxi Fu. **Liuyang CDC:** Xin Xu, Hao Zhang, Jianwei Chen,Yuan Peng, Libo Zhang, Chan Qu.

# Supplemental Method

## CHEERS 2022 Checklist

| Topic | No. | Item | Section, Paragraph, Page Number |
| --- | --- | --- | --- |
| **Title** |  |  |  |
|  | 1 | Identify the study as an economic evaluation and specify the interventions being compared. | Title, Page 1 |
| **Abstract** |  |  |  |
|  | 2 | Provide a structured summary that highlights context, key methods, results, and alternative analyses. | Abstract, Page 2-3 |
| **Introduction** |  |  |  |
| Background and  objectives | 3 | Give the context for the study, the study question, and its practical relevance for decision making in policy or practice. | Background, paragraphs 1-4, Page 4-5 |
| **Methods** |  |  |  |
| Health economic  analysis plan | 4 | Indicate whether a health economic analysis plan was developed and where available. | Methods, paragraphs 1-3, Page 6-7 |
| Study population | 5 | Describe characteristics of the study population (such as age range, demographics, socioeconomic, or clinical characteristics). | Methods, paragraphs 4-5, Page 7-8 |
| Setting and location | 6 | Provide relevant contextual information that may influence findings. | Methods, paragraphs 4-5, Page 7-8 |
| Comparators | 7 | Describe the interventions or strategies being compared and why chosen. | Methods, paragraph 8, Page 9 |
| Perspective | 8 | State the perspective(s) adopted by the study and why chosen. | Methods, paragraph 1, Page 6 |
| Time horizon | 9 | State the time horizon for the study and why appropriate. | Methods, paragraph 1, Page 6 |
| Discount rate | 10 | Report the discount rate(s) and reason chosen. | Methods, paragraph 18, Page 13 |
| Selection of outcomes | 11 | Describe what outcomes were used as the measure(s) of benefit(s) and harm(s). | Methods, paragraph 17, Page 12 |
| Measurement of  outcomes | 12 | Describe how outcomes used to capture benefit(s) and harm(s) were measured. | Methods, paragraph 17, Page 12 |
| Valuation of outcomes | 13 | Describe the population and methods used to measure and value outcomes. | Methods, paragraph 17, Page 12 |
| Measurement and  valuation of resources  and costs | 14 | Describe how costs were valued. | Methods, paragraphs 13-15, Page 11-12 |
| Currency, price date,  and conversion | 15 | Report the dates of the estimated resource quantities and unit costs, plus the currency and year of conversion. | Methods, paragraphs 13-15, Page 11-12 |
| Rationale and  description of model | 16 | If modelling is used, describe in detail and why used. Report if the model is publicly available and where it can be accessed. | Methods, paragraphs 1-3, Page 6-7 |
| Analytics and  assumptions | 17 | Describe any methods for analysing or statistically transforming data, any extrapolation methods, and approaches for validating any model used. | Methods, paragraphs 15-16, Page 12; Results, paragraph 1, Page 14 |
| Characterising  heterogeneity | 18 | Describe any methods used for estimating how the results of the study vary for subgroups. | Methods, paragraph 20, Page 13-14 |
| Characterising  distributional effects | 19 | Describe how impacts are distributed across different individuals or adjustments made to reflect priority populations. | Methods, paragraph 19, Page 13; Table 1 |
| Characterising  uncertainty | 20 | Describe methods to characterise any sources of uncertainty in the analysis. | Methods, paragraph 19, Page 13; Table 1 |
| Approach to  engagement with  patients and others  affected by the study | 21 | Describe any approaches to engage patients or service recipients, the general public, communities, or stakeholders (such as clinicians or payers) in the design of the study. | NA |
| **Results** |  |  |  |
| Study parameters | 22 | Report all analytic inputs (such as values, ranges, references) including uncertainty or distributional assumptions. | Table 1 |
| Summary of main  results | 23 | Report the mean values for the main categories of costs and outcomes of interest and summarise them in the most appropriate overall measure. | Results, paragraph 3, 8-9, Page 14-16; Tables 2 and 4 |
| Effect of uncertainty | 24 | Describe how uncertainty about analytic judgments, inputs, or projections affect findings. Report the effect of choice of discount rate and time horizon, if applicable. | Results, paragraphs 4-5, Page 15; Figure 2 |
| Effect of engagement  with patients and  others affected by the  study | 25 | Report on any difference patient/service recipient, general public, community, or stakeholder involvement made to the approach or findings of the study | NA |
| **Discussion** |  |  |  |
| Study findings,  limitations,  generalisability, and  current knowledge | 26 | Report key findings, limitations, ethical or equity considerations not captured, and how these could affect patients, policy, or practice. | Discussion, Page 17-22 |
| **Other relevant**  **information** |  |  |  |
| Source of funding | 27 | Describe how the study was funded and any role of the funder in the identification, design, conduct, and reporting of the analysis. | Funding, Page 25 |
| Conflicts of interest | 28 | Report authors conflicts of interest according to journal or International Committee of Medical Journal Editors requirements. | Competing interests, Page 26 |

# Supplemental Figures

1. Markov decision tree for health states


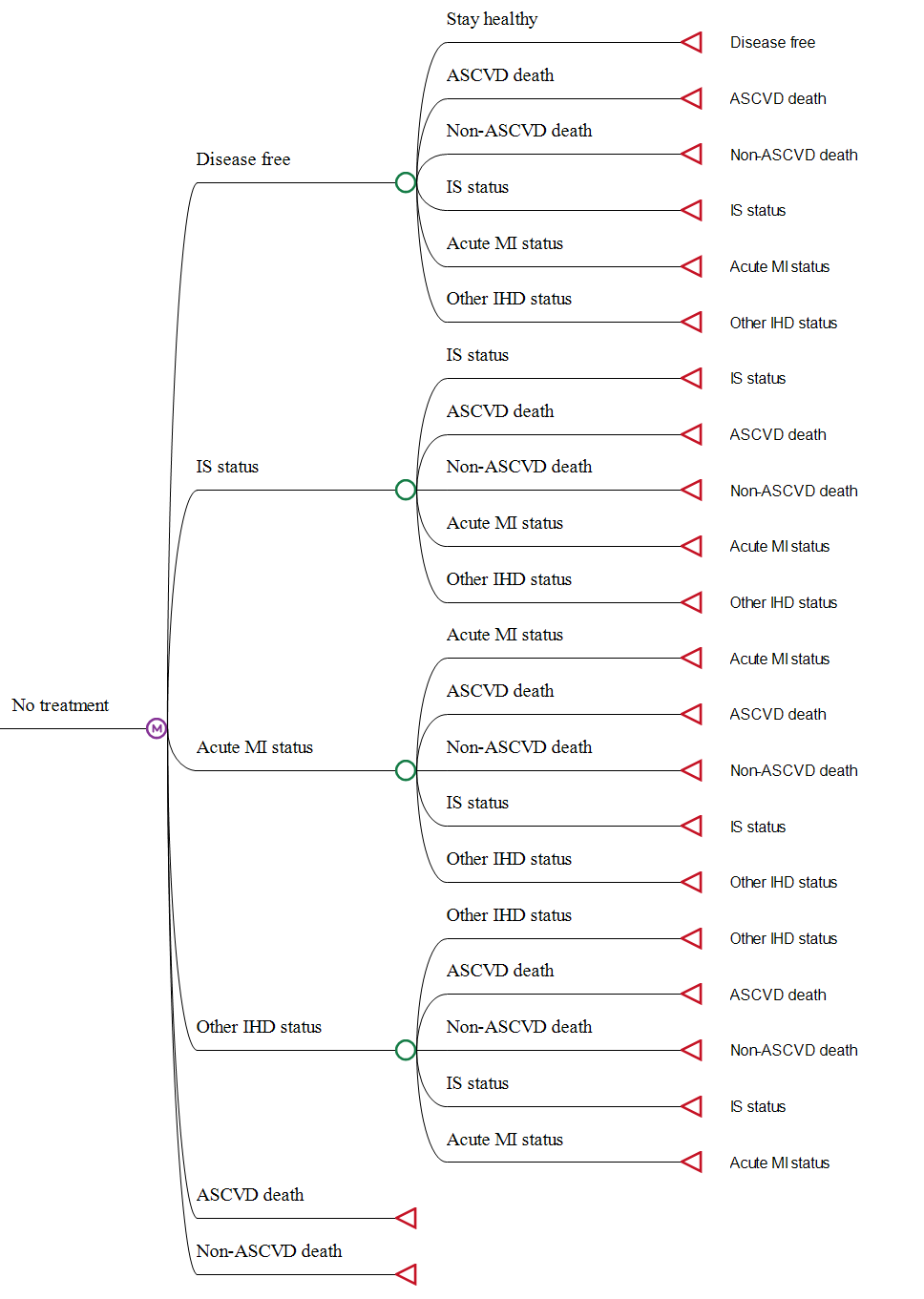


1. Markov decision tree for interventions


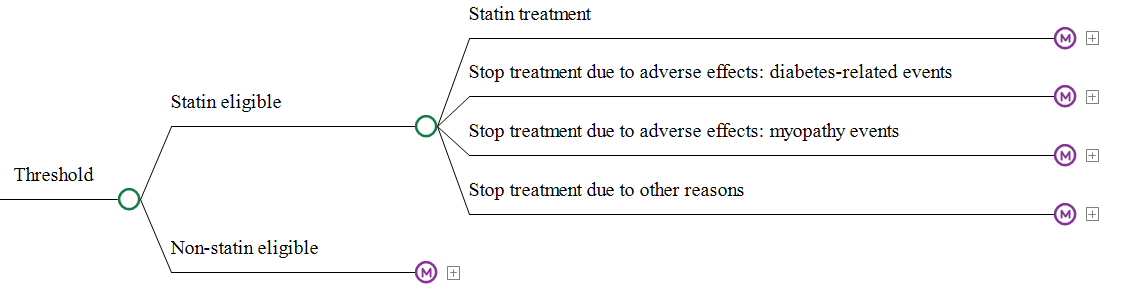


## Figure S1. Structure of the Markov model

IS: ischemic stroke; MI: myocardial infarction; IHD: ischemic heart disease; ASCVD: atherosclerotic cardiovascular disease.


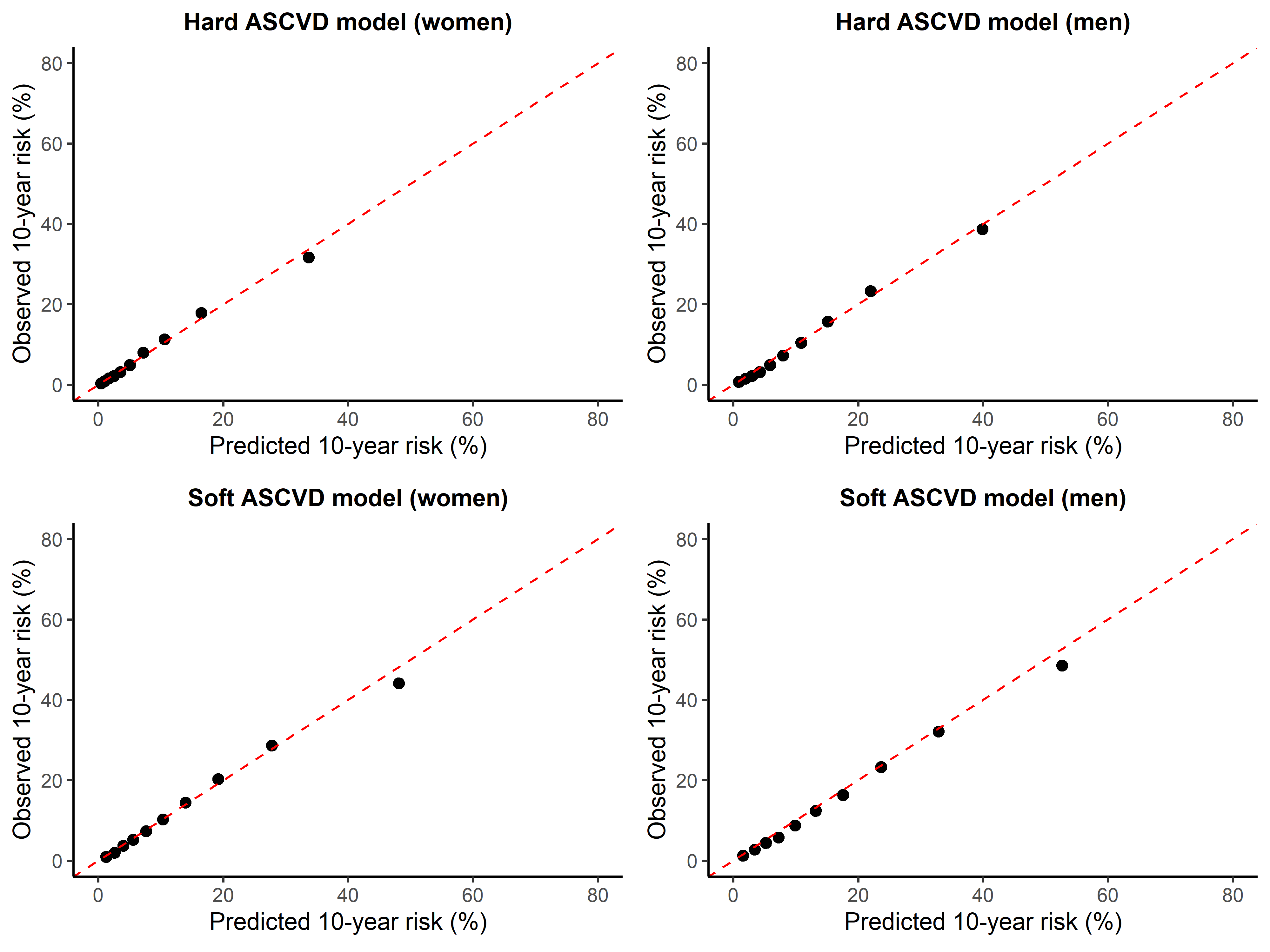


## Figure S2. Calibration plots for CKB hard and soft ASCVD models after recalibration.

CKB: China Kadoorie Biobank; ASCVD: atherosclerotic cardiovascular disease.


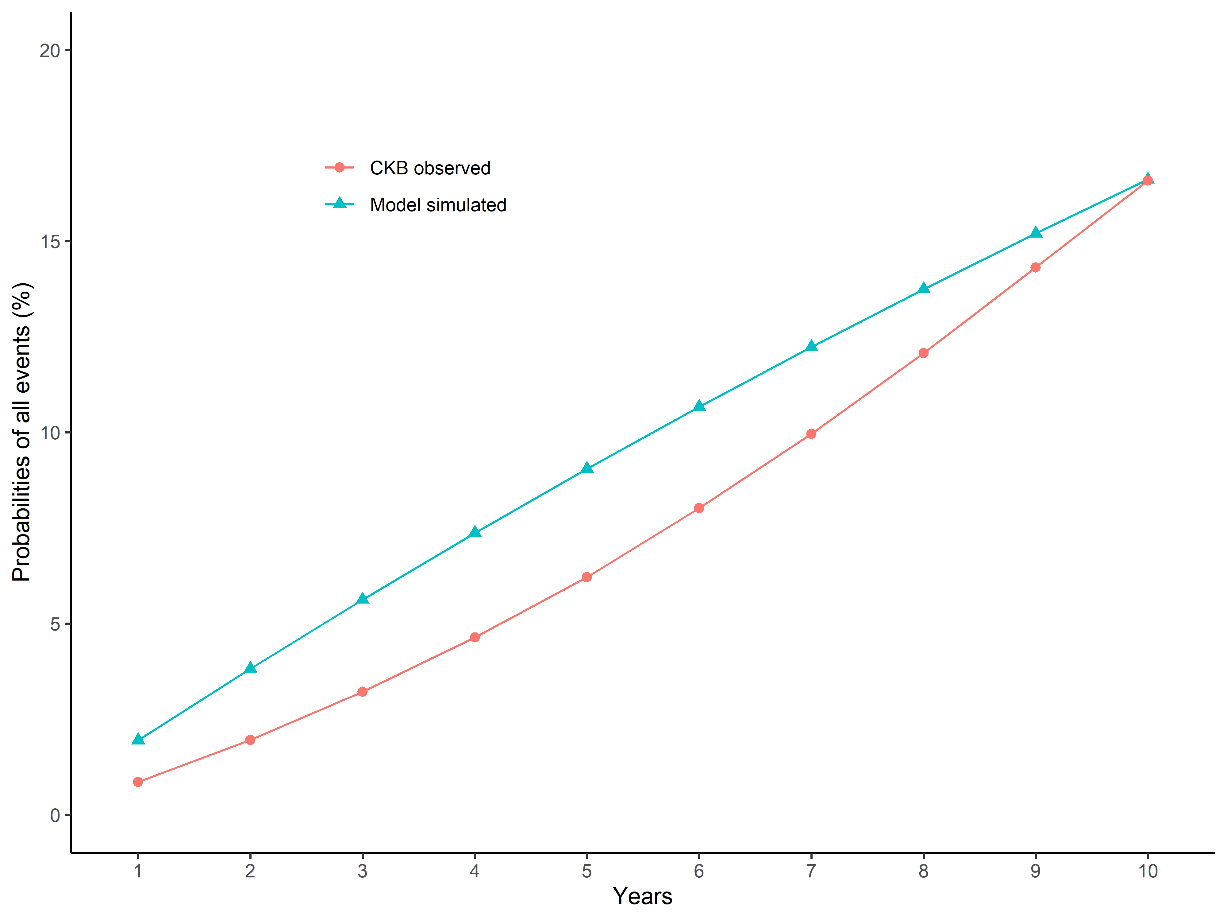


## Figure S3. Observed vs. simulated probabilities of all events over 10 years

CKB: China Kadoorie Biobank.

All events included nonfatal atherosclerotic cardiovascular disease events and all fatal events.


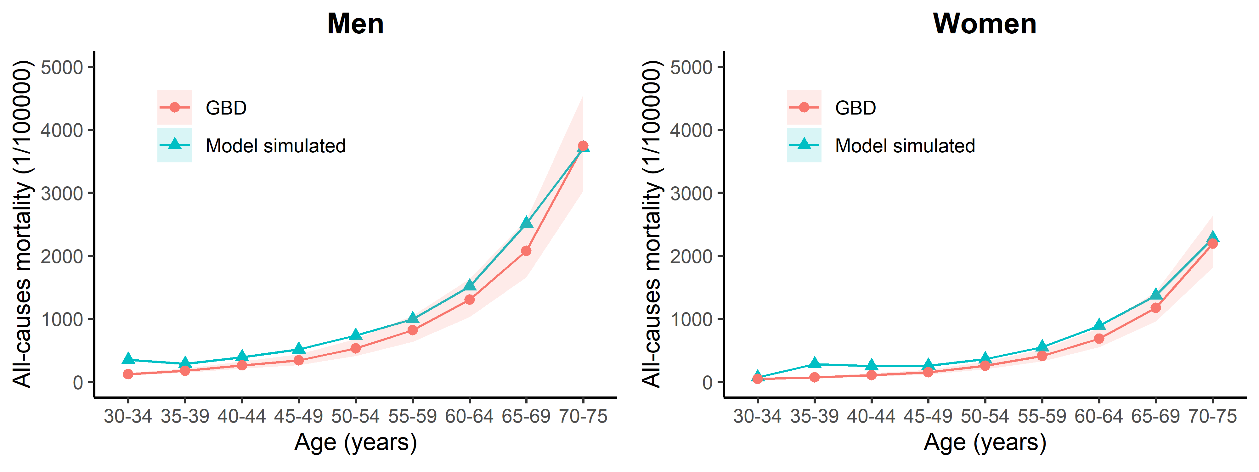


## Figure S4. GBD vs. simulated age-sex-specific all-cause mortality (1/100000)

GBD: Global Burden of Disease.

Red shades represent the 95% confidence interval of the mortality from the GBD study.

**
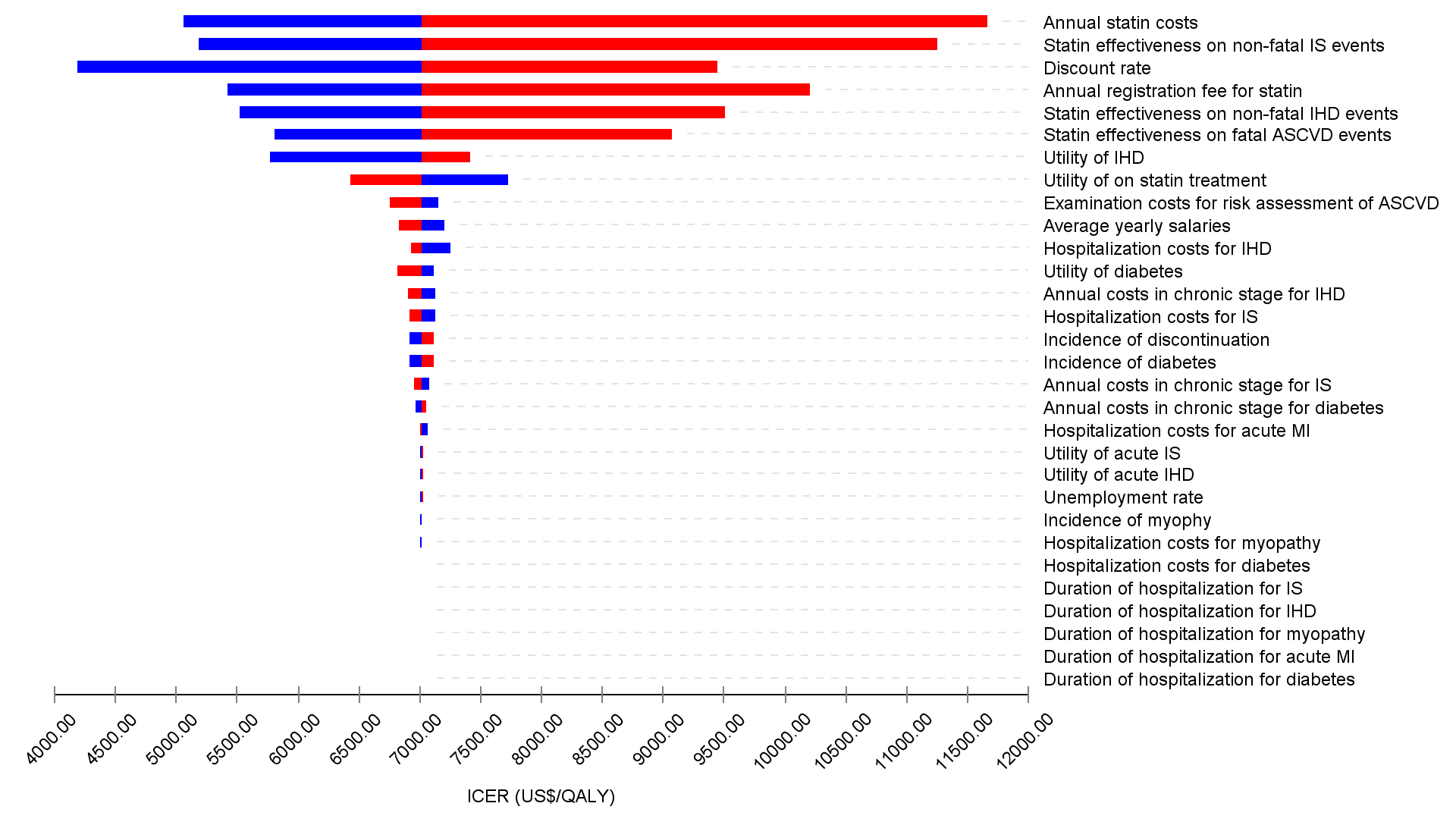
**

## Figure S5. One-way sensitivity analyses for soft ASCVD model threshold of 18% vs. 19%

ASCVD: atherosclerotic cardiovascular disease; IS: ischemic stroke; IHD: ischemic heart disease; GDP: gross domestic product.

Blue and red bars indicate parameter ranges lower and higher than the base-case values shown in Table 1, respectively.

**
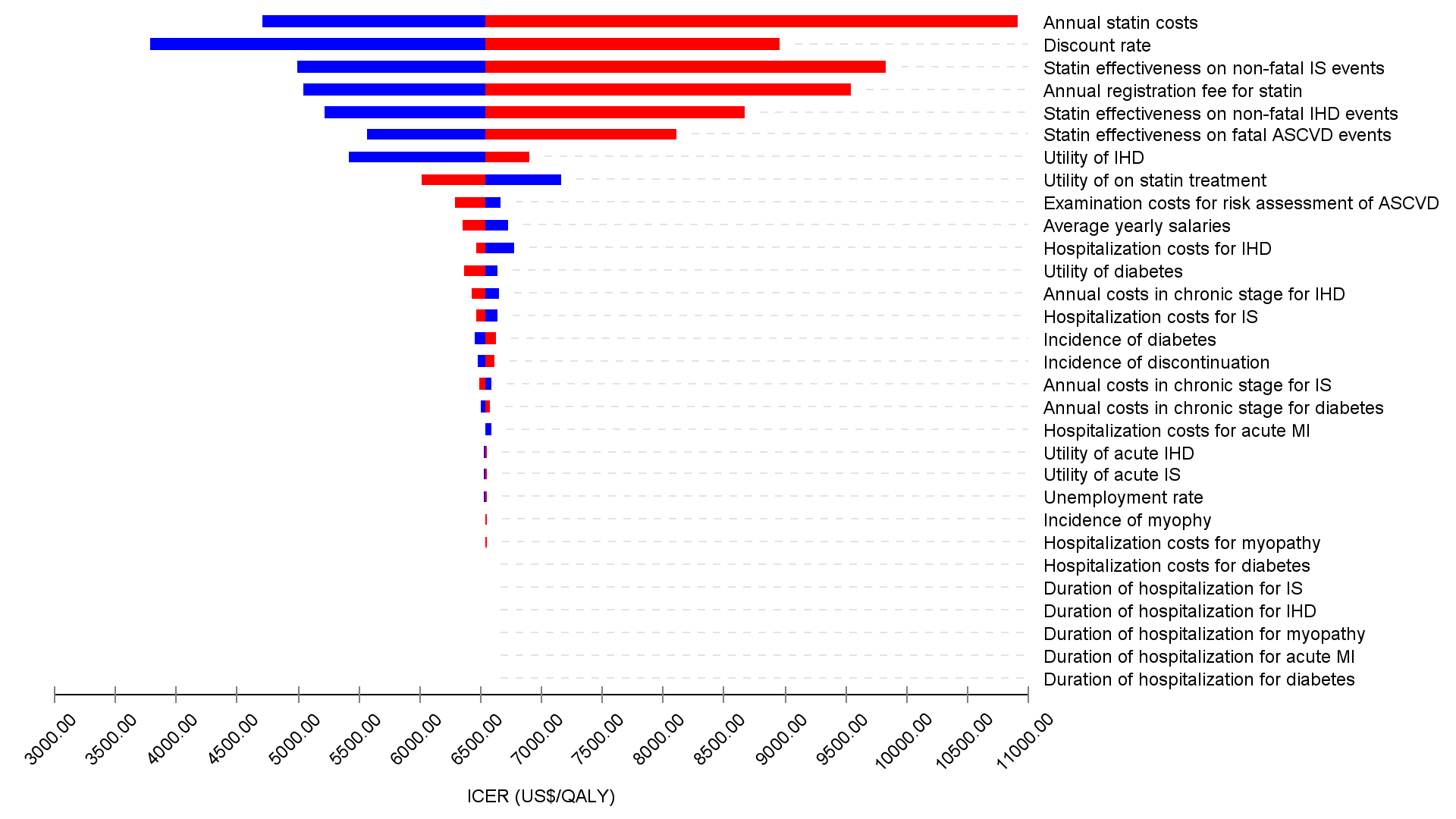
**

## Figure S6. One-way sensitivity analyses for hard ASCVD model threshold of 10% vs. 11%

ASCVD: atherosclerotic cardiovascular disease; IS: ischemic stroke; IHD: ischemic heart disease; GDP: gross domestic product.

Blue and red bars indicate parameter ranges lower and higher than the base-case values shown in Table 1, respectively.


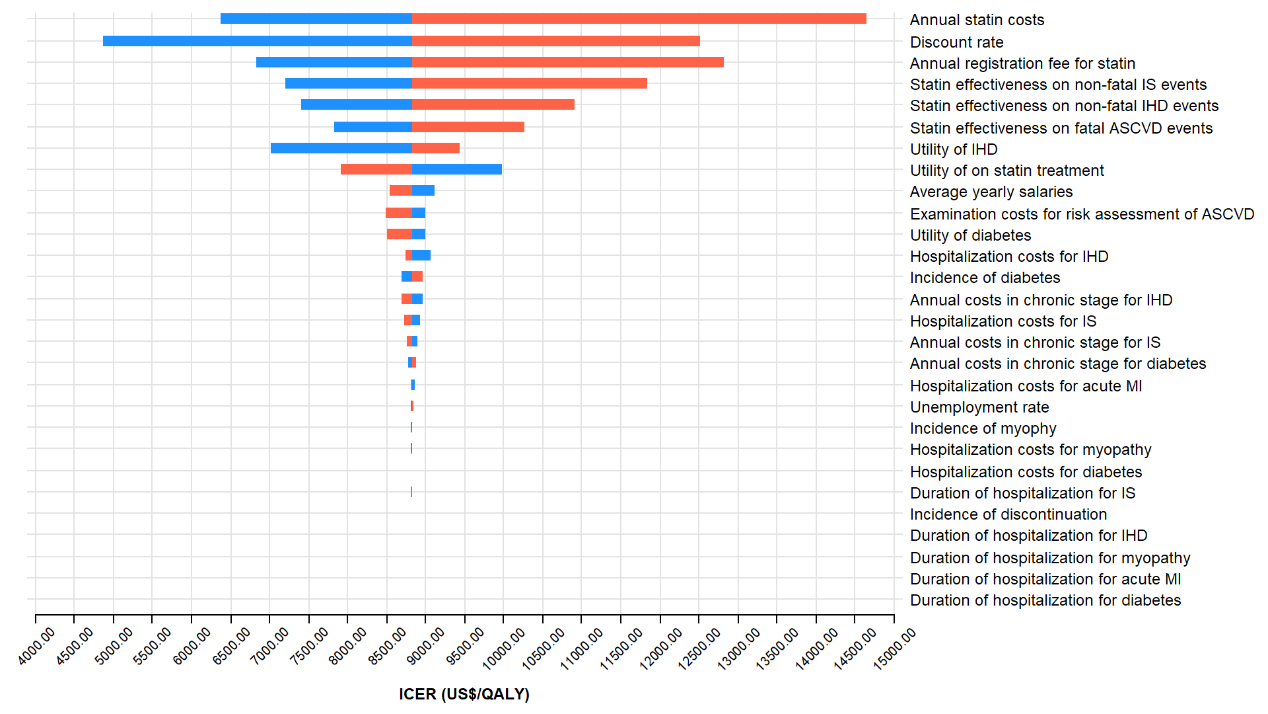


## Figure S7. One-way sensitivity analyses for soft ASCVD model threshold of 13% vs. 14% in population aged 30-59 years

ASCVD: atherosclerotic cardiovascular disease; IS: ischemic stroke; IHD: ischemic heart disease; GDP: gross domestic product.

Blue and red bars indicate parameter ranges lower and higher than the base-case values shown in Table 1, respectively.


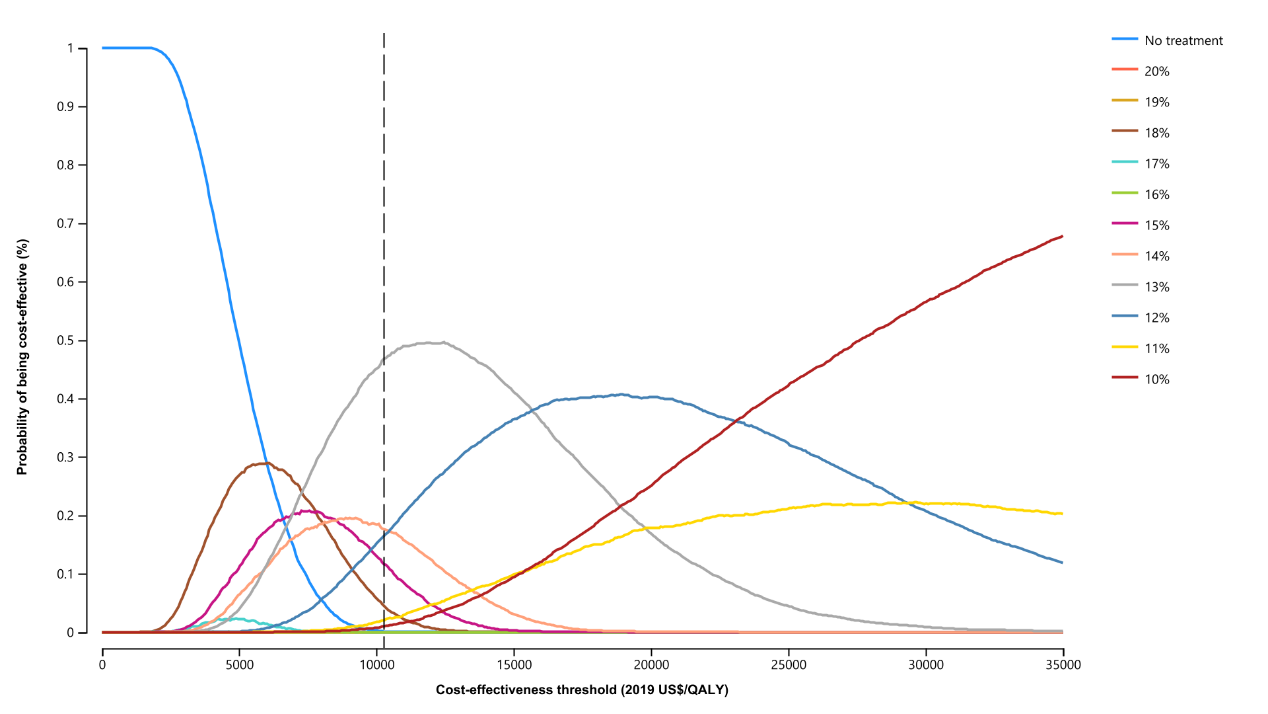


## Figure S8. Cost-utility acceptability curves for soft ASCVD threshold strategies in population aged 30-59 years

ASCVD: atherosclerotic cardiovascular disease; QALYs: quality-adjusted life year.

Dashed lines indicate the GDP per capita in 2019.


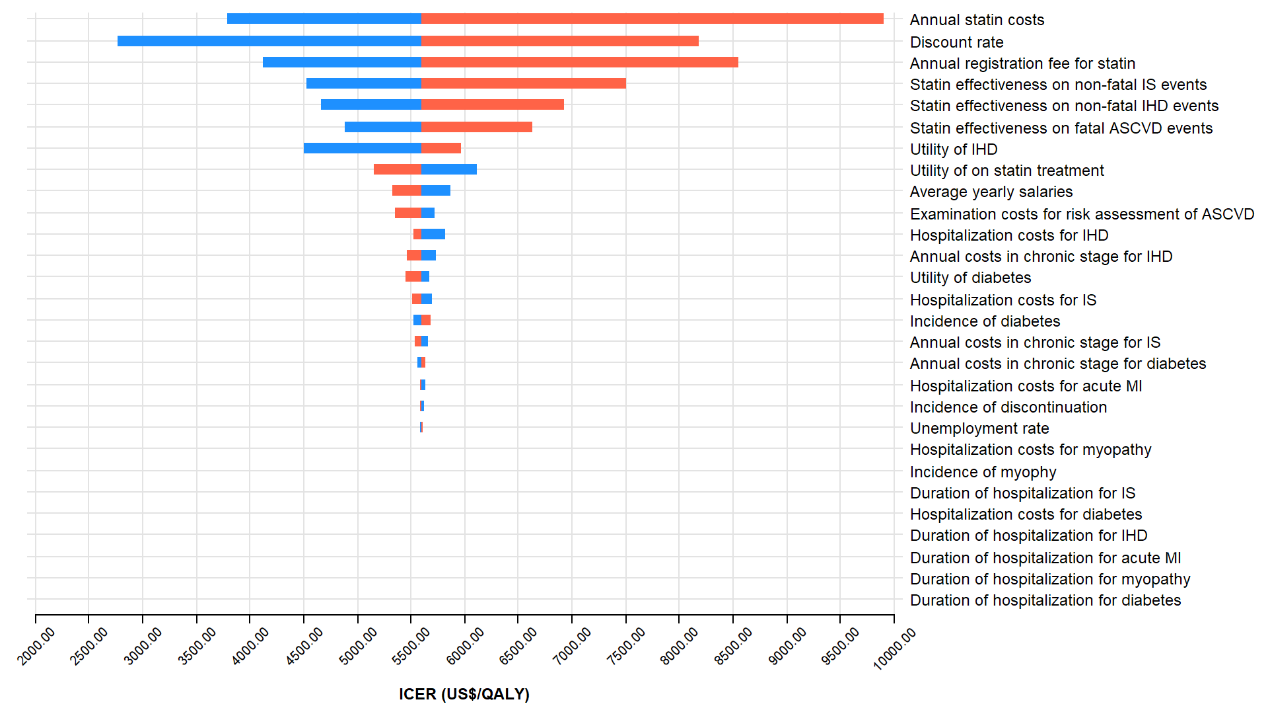


## Figure S9. One-way sensitivity analyses for hard ASCVD model threshold of 9% vs. 10% in population aged 30-59 years

ASCVD: atherosclerotic cardiovascular disease; IS: ischemic stroke; IHD: ischemic heart disease; GDP: gross domestic product.

Blue and red bars indicate parameter ranges lower and higher than the base-case values shown in Table 1, respectively.


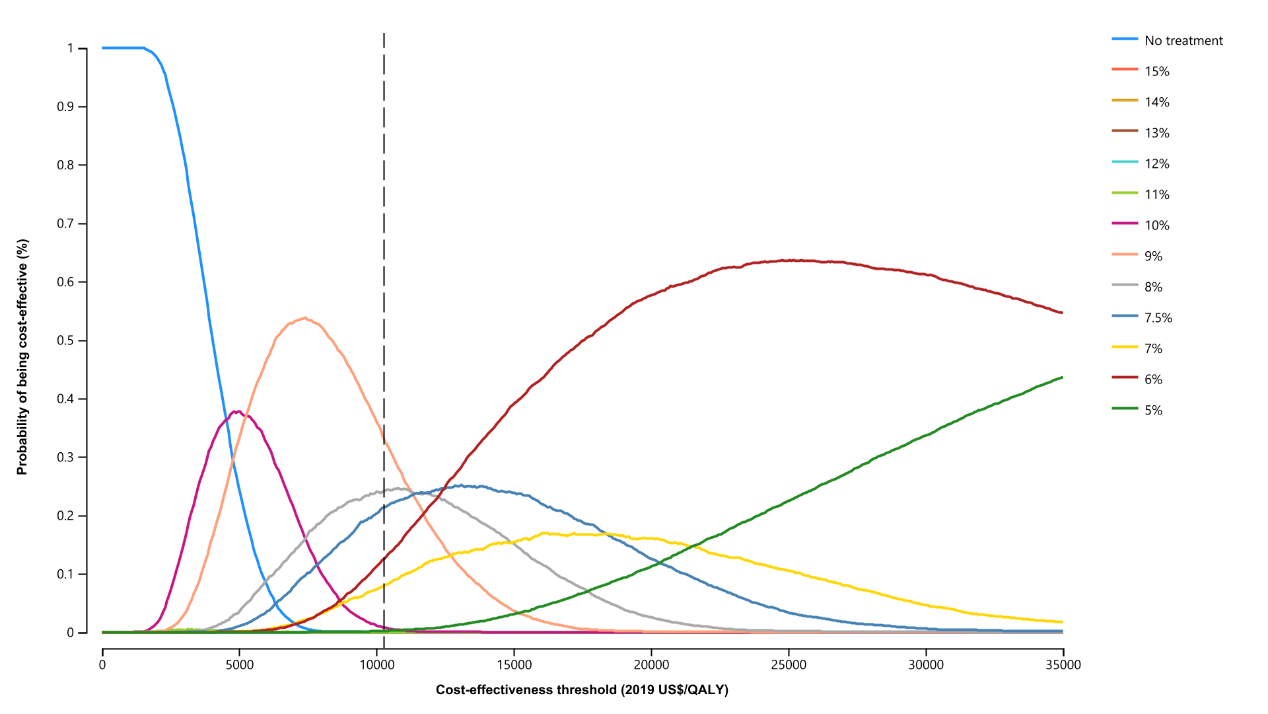


## Figure S10. Cost-utility acceptability curves for hard ASCVD threshold strategies in population aged 30-59 years

ASCVD: atherosclerotic cardiovascular disease; QALYs: quality-adjusted life year.

Dashed lines indicate the GDP per capita in 2019.


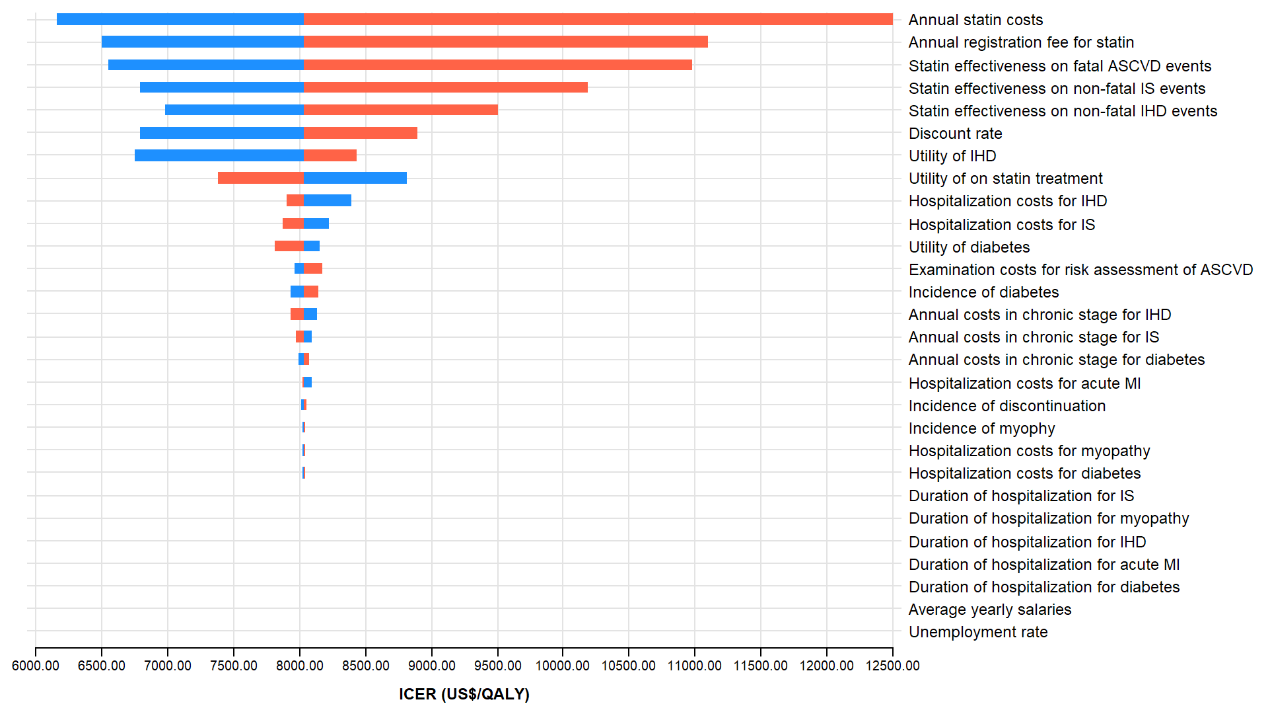


## Figure S11. One-way sensitivity analyses for soft ASCVD model threshold of 20% vs. no treatment in population aged 60-75 years

ASCVD: atherosclerotic cardiovascular disease; IS: ischemic stroke; IHD: ischemic heart disease; GDP: gross domestic product.

Blue and red bars indicate parameter ranges lower and higher than the base-case values shown in Table 1, respectively.


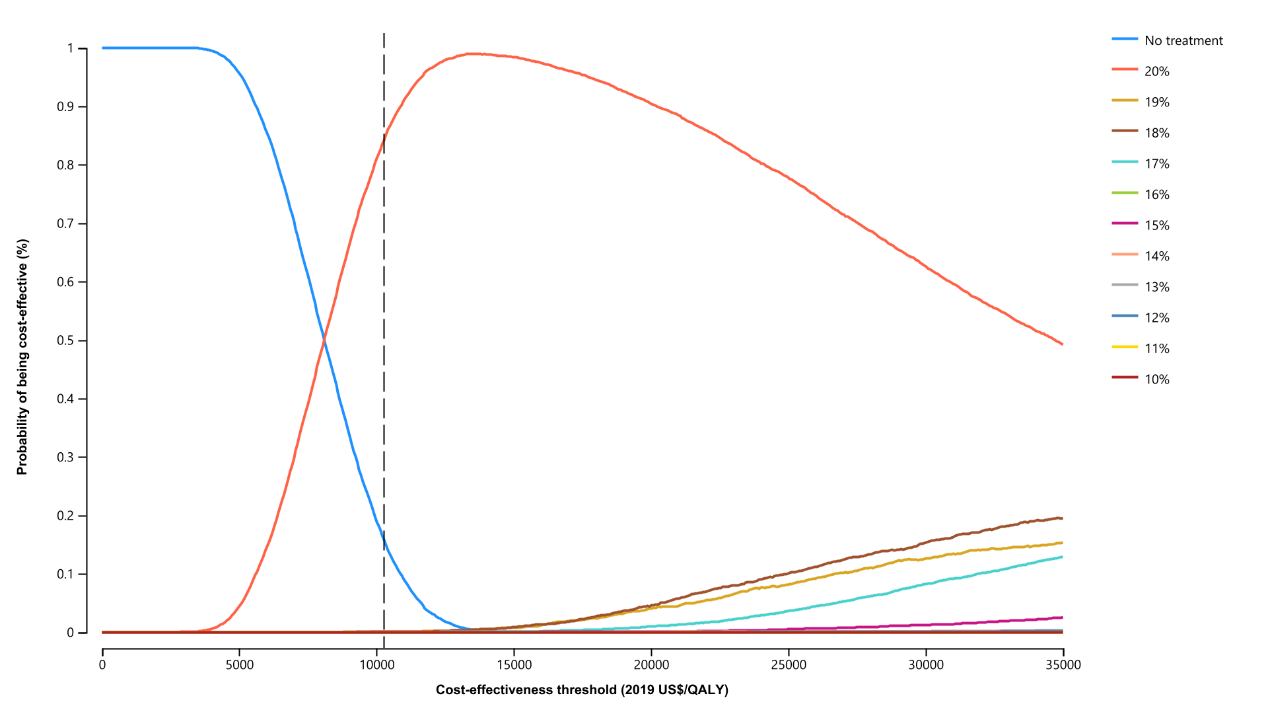


## Figure S12. Cost-utility acceptability curves for soft ASCVD threshold strategies in population aged 60-75 years

ASCVD: atherosclerotic cardiovascular disease; QALYs: quality-adjusted life year.

Dashed lines indicate the GDP per capita in 2019.


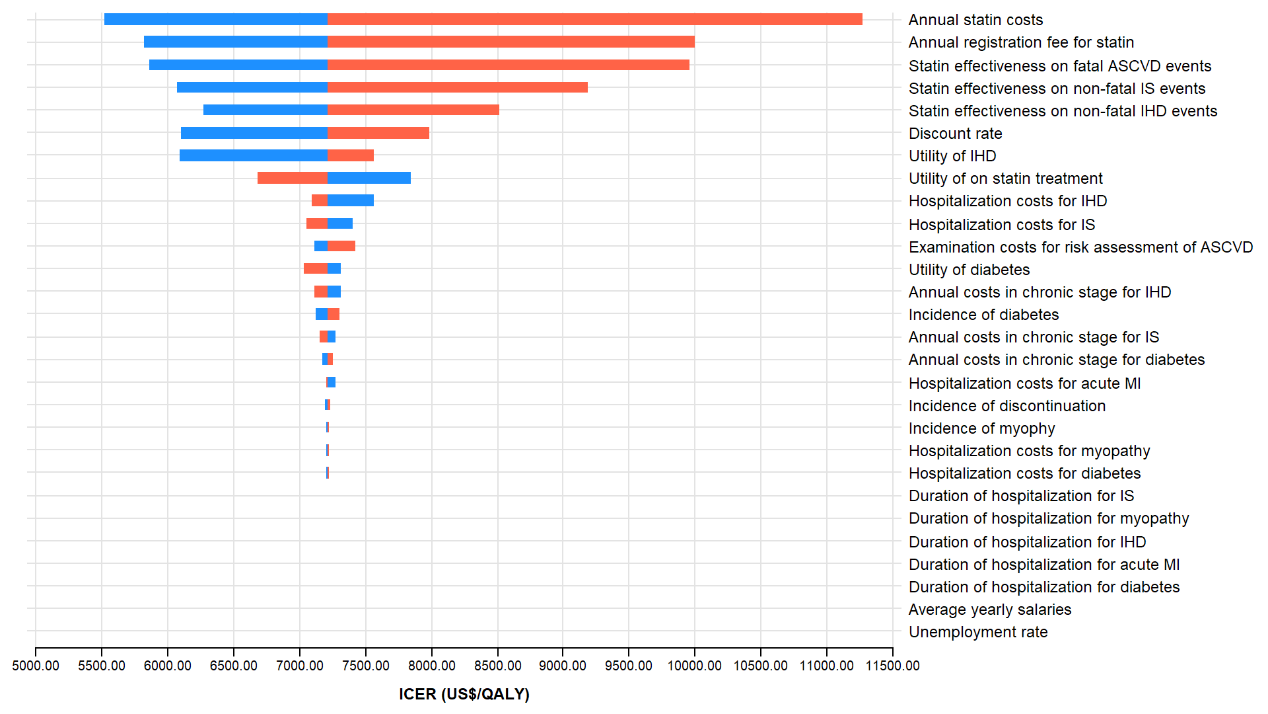


## Figure S13. One-way sensitivity analyses for hard ASCVD model threshold of 15% vs. no treatment in population aged 60-75 years

ASCVD: atherosclerotic cardiovascular disease; IS: ischemic stroke; IHD: ischemic heart disease; GDP: gross domestic product.

Blue and red bars indicate parameter ranges lower and higher than the base-case values shown in Table 1, respectively.


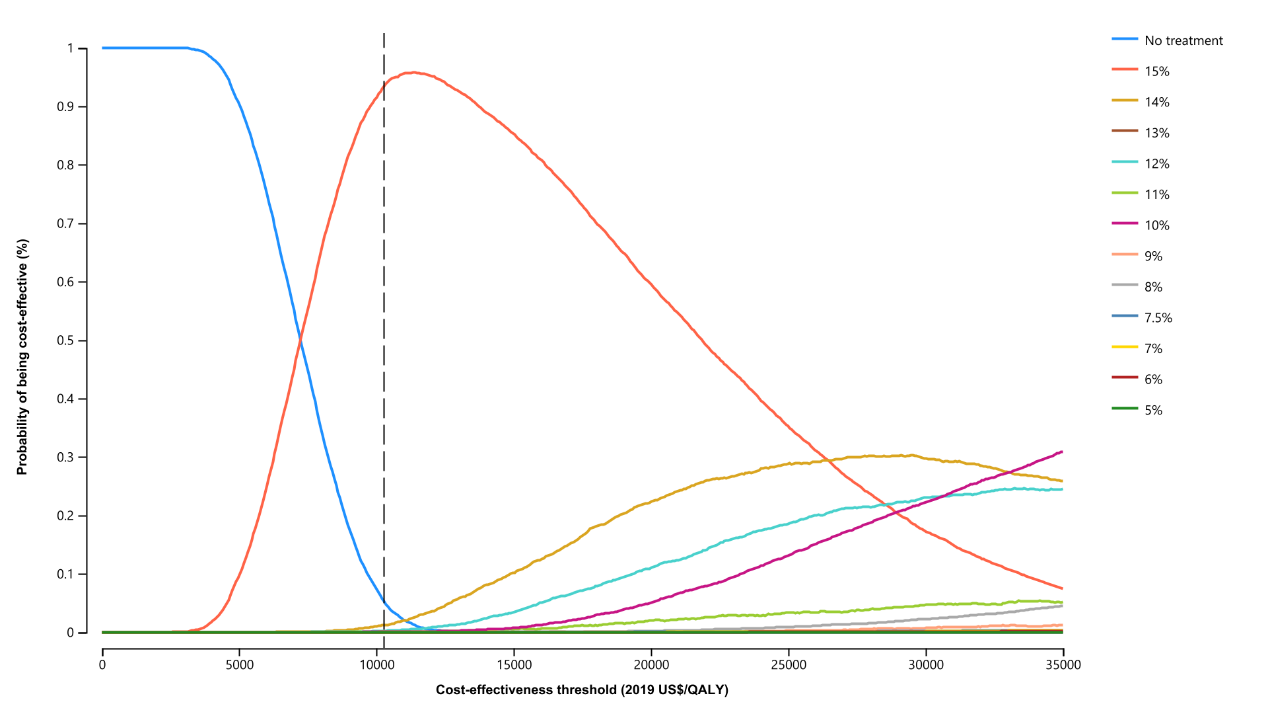


## Figure S14. Cost-utility acceptability curves for hard ASCVD threshold strategies in population aged 60-75 years

ASCVD: atherosclerotic cardiovascular disease; QALYs: quality-adjusted life year.

Dashed lines indicate the GDP per capita in 2019.

# Supplemental Tables

## Table S1. Disease progression inputs used in the cost-utility model

| Transition^a^ | Value |
| --- | --- |
| From Acute MI status to |  |
| Other IHD | 0.1468 |
| IS | 0.0293 |
| ASCVD death | 0.0327 |
| Death from other causes | 0.0242 |
| From other IHD status to |  |
| Acute MI | 0.0048 |
| IS | 0.0317 |
| ASCVD death | 0.0132 |
| Death from other causes | 0.0232 |
| From IS status to |  |
| Acute MI | 0.0023 |
| Other IHD | 0.0281 |
| ASCVD death | 0.0119 |
| Death from other causes | 0.0293 |

MI: myocardial infarction; IHD: ischemic heart disease; IS: ischemic stroke; ASCVD: atherosclerotic cardiovascular disease.

^a^ The transition probabilities from disease-free to other states calculated for each ASCVD risk threshold strategy are not shown.

## Table S2. Baseline characteristics of participants with soft or hard ASCVD outcomes occurred during follow-up in CKB study

|  | Soft ASCVD outcomes | Hard ASCVD outcomes |
| --- | --- | --- |
| Number of participants | 82,575 | 55,704 |
| Women | 57.2 | 54.1 |
| Urban area | 50.1 | 49.7 |
| Age, years | 58.3 (51.1-66.4) | 59.4 (52.1-67.2) |
| Current daily smoker | 25.1 | 26.6 |
| Taking antihypertensive treatment | 21.5 | 22.9 |
| Systolic blood pressure, mmHg | 136.0 (122.5-153.5) | 138.5 (124.0-156.0) |
| Diastolic blood pressure, mmHg | 79.5 (72.0-87.5) | 80.0 (72.0-88.5) |
| Waist circumference, cm | 82.4 (75.4-89.5) | 82.5 (75.6-89.6) |
| Self-reported diabetes | 6.2 | 6.7 |

ASCVD: atherosclerotic cardiovascular disease; CKB: China Kadoorie Biobank.

Data are percentage or median (25-75^th^ percentile) unless otherwise specified.

**Table S3. Baseline characteristics of high-risk population defined by optimal thresholds for soft and hard ASCVD models**

|  | Soft ASCVD model  Risk > 18% | Hard ASCVD model  Risk > 10% |
| --- | --- | --- |
| Number of participants | 143,456 | 149,045 |
| Women | 53.2 | 50.2 |
| Urban area | 49.5 | 48.8 |
| Age, years | 62.8 (56.2-68.5) | 62.4 (55.9-68.4) |
| Current daily smoker | 27.3 | 29.1 |
| Taking antihypertensive treatment | 26.7 | 26.0 |
| Systolic blood pressure, mmHg | 141.0 (127.0-158.0) | 142.0 (128.0-158.5) |
| Diastolic blood pressure, mmHg | 80.5 (72.5-89.0) | 81.0 (73.0-89.5) |
| Waist circumference, cm | 83.2 (76.1-90.2) | 83.0 (76.0-90.0) |
| Self-reported diabetes | 7.3 | 7.1 |

ASCVD: atherosclerotic cardiovascular disease; CKB: China Kadoorie Biobank.

Data are percentage or median (25-75^th^ percentile) unless otherwise specified.

## Table S4. Cost-utility of ASCVD threshold strategies for hard ASCVD model by age

| ASCVD risk threshold (%) | 30-59 years | | | |  | 60-75 years | | | |
| --- | --- | --- | --- | --- | --- | --- | --- | --- | --- |
|  | Statin  eligible (%) | Costs  (2019 US$) | QALYs | ICER  (US$/QALY) |  | Statin eligible (%) | Costs  (2019 US$) | QALYs | ICER  (US$/QALY) |
| No treatment | 0 | 2642.41 | 15.3082 | Ref |  | 0 | 592.76 | 7.7321 | Ref |
| 15 | 6.8 | 2730.03 | 15.3212 | Extended dominance^a^ |  | 62.9 | 942.09 | 7.7805 | 7213.30 |
| 14 | 7.8 | 2731.47 | 15.3244 | 452.07 |  | 66.6 | 966.57 | 7.7817 | 20878.43 |
| 13 | 9.0 | 2735.36 | 15.3276 | 1225.98 |  | 70.4 | 991.81 | 7.7825 | Extended dominance^a^ |
| 12 | 10.5 | 2741.83 | 15.3308 | 1993.46 |  | 74.3 | 1017.97 | 7.7835 | 27433.99 |
| 11 | 12.2 | 2752.17 | 15.3342 | 3059.07 |  | 78.1 | 1043.91 | 7.7843 | 35954.05 |
| **10** | 14.3 | **2766.41** | **15.3379** | **4185.19** |  | 81.7 | **1068.76** | **7.7849** | **38453.64** |
| 9 | 16.9 | 2786.95 | 15.3415 | 5597.65 |  | 85.3 | 1094.46 | 7.7852 | 87487.43 |
| 8 | 20.0 | 2815.83 | 15.3448 | 8783.87 |  | 88.6 | 1117.90 | 7.7854 | 102184.90 |
| 7.5 | 21.9 | 2833.89 | 15.3465 | 10766.20 |  | 90.2 | 1129.33 | 7.7854 | 490103.05 |
| 7 | 23.9 | 2854.82 | 15.3481 | 13446.47 |  | 91.8 | 1141.20 | 7.7855 | 820026.10 |
| 6 | 28.5 | 2905.53 | 15.3513 | 15581.01 |  | 94.6 | 1162.37 | 7.7854 | Dominated^b^ |
| 5 | 34.3 | 2976.19 | 15.3533 | 35138.52 |  | 97.0 | 1180.71 | 7.7852 | Dominated^b^ |

ASCVD: atherosclerotic cardiovascular disease; QALYs: quality-adjusted life year; ICER: incremental cost-effectiveness ratio.

^a^ Extended dominance indicates larger ICER than a more effective strategy.

^b^ Dominated indicates higher cost and lower QALYs than the comparator.

## Table S5. Cost-utility of ASCVD threshold strategies for soft ASCVD model by region

| ASCVD risk threshold (%) | Urban | | | |  | Rural | | | |
| --- | --- | --- | --- | --- | --- | --- | --- | --- | --- |
|  | Statin  eligible (%) | Costs  (2019 US$) | QALYs | ICER  (US$/QALY) |  | Statin eligible (%) | Costs  (2019 US$) | QALYs | ICER  (US$/QALY) |
| No treatment | 0 | 2931.00 | 14.0321 | Ref |  | 0 | 2862.47 | 13.8604 | Ref |
| 20 | 28.0 | 3052.06 | 14.1099 | 1554.75 |  | 23.4 | 3059.90 | 13.8991 | 5111.70 |
| 19 | 29.5 | 3060.98 | 14.1130 | 2915.72 |  | 24.9 | 3072.37 | 13.9011 | 6053.32 |
| **18** | **31.1** | **3073.05** | **14.1151** | **5709.01** |  | **26.5** | **3086.88** | **13.9028** | **8868.02** |
| 17 | 32.8 | 3089.22 | 14.1165 | 11471.56 |  | 28.2 | 3104.25 | 13.9038 | 17040.40 |
| 16 | 34.5 | 3106.56 | 14.1176 | Extended dominance^a^ |  | 30.0 | 3124.46 | 13.9042 | Extended dominance^a^ |
| 15 | 36.4 | 3123.94 | 14.1192 | 12895.07 |  | 32.0 | 3145.47 | 13.9050 | 26011.88 |
| 14 | 38.5 | 3146.78 | 14.1200 | Extended dominance^a^ |  | 34.2 | 3169.30 | 13.9058 | 32532.07 |
| 13 | 40.7 | 3170.04 | 14.1210 | 25829.35 |  | 36.6 | 3197.67 | 13.9059 | 420346.94 |
| 12 | 43.0 | 3196.37 | 14.1216 | 41171.39 |  | 39.2 | 3231.23 | 13.9048 | Dominated^b^ |
| 11 | 45.7 | 3230.34 | 14.1210 | Dominated^b^ |  | 42.1 | 3269.16 | 13.9033 | Dominated^b^ |
| 10 | 48.6 | 3267.96 | 14.1200 | Dominated^b^ |  | 45.2 | 3311.69 | 13.9015 | Dominated^b^ |

ASCVD: atherosclerotic cardiovascular disease; QALYs: quality-adjusted life year; ICER: incremental cost-effectiveness ratio.

^a^ Extended dominance indicates larger ICER than a more effective strategy.

^b^ Dominated indicates higher cost and lower QALYs than the comparator.

## Table S6. Cost-utility of ASCVD threshold strategies for hard ASCVD model by region

| ASCVD risk threshold (%) | Urban | | | |  | Rural | | | |
| --- | --- | --- | --- | --- | --- | --- | --- | --- | --- |
|  | Statin  eligible (%) | Costs  (2019 US$) | QALYs | ICER  (US$/QALY) |  | Statin eligible (%) | Costs  (2019 US$) | QALYs | ICER  (US$/QALY) |
| No treatment | 0 | 2934.80 | 14.0326 | Ref |  | 0 | 2873.01 | 13.8546 | Ref |
| 15 | 22.3 | 3031.08 | 14.0949 | Extended dominance^a^ |  | 17.6 | 3025.99 | 13.8843 | Extended dominance^a^ |
| 14 | 23.9 | 3036.70 | 14.0999 | 1515.77 |  | 19.3 | 3035.04 | 13.8888 | 1991.99 |
| 13 | 25.7 | 3045.42 | 14.1041 | 2046.48 |  | 21.1 | 3047.45 | 13.8925 | 3340.78 |
| 12 | 27.6 | 3056.59 | 14.1081 | 2800.04 |  | 23.2 | 3061.47 | 13.8969 | 4456.86 |
| 11 | 29.8 | 3070.83 | 14.1122 | 3510.63 |  | 25.4 | 3081.14 | 13.8996 | Extended dominance^a^ |
| **10** | **32.2** | **3090.85** | **14.1151** | **6796.39** |  | **27.9** | **3102.27** | **13.9029** | **6747.36** |
| 9 | 35.0 | 3115.17 | 14.1180 | 8289.81 |  | 30.7 | 3132.53 | 13.9042 | 24463.80 |
| 8 | 38.0 | 3146.27 | 14.1198 | 17548.48 |  | 33.9 | 3168.13 | 13.9050 | 43923.58 |
| 7.5 | 39.7 | 3165.09 | 14.1200 | Extended dominance^a^ |  | 35.8 | 3189.61 | 13.9051 | 199307.96 |
| 7 | 41.5 | 3185.65 | 14.1204 | 50977.47 |  | 37.7 | 3214.12 | 13.9046 | Dominated^b^ |
| 6 | 45.6 | 3231.12 | 14.1213 | 57824.87 |  | 42.1 | 3271.49 | 13.9027 | Dominated^b^ |
| 5 | 50.3 | 3291.13 | 14.1202 | Dominated^b^ |  | 47.4 | 3345.77 | 13.8982 | Dominated^b^ |

ASCVD: atherosclerotic cardiovascular disease; QALYs: quality-adjusted life year; ICER: incremental cost-effectiveness ratio.

^a^ Extended dominance indicates larger ICER than a more effective strategy.

^b^ Dominated indicates higher cost and lower QALYs than the comparator.

## Table S7. Cost-utility of ASCVD threshold strategies for soft ASCVD model by sex

| ASCVD risk threshold (%) | Female | | | |  | Male | | | |
| --- | --- | --- | --- | --- | --- | --- | --- | --- | --- |
|  | Statin  eligible (%) | Costs  (2019 US$) | QALYs | ICER  (US$/QALY) |  | Statin eligible (%) | Costs  (2019 US$) | QALYs | ICER  (US$/QALY) |
| No treatment | 0 | 2530.40 | 14.1591 | Ref |  | 0 | 3260.93 | 13.7324 | Ref |
| 20 | 24.3 | 2679.44 | 14.2187 | 2501.35 |  | 26.4 | 3434.15 | 13.7835 | 3387.22 |
| 19 | 25.8 | 2688.73 | 14.2216 | 3185.09 |  | 28.0 | 3446.55 | 13.7856 | 5998.58 |
| **18** | **27.3** | **2701.20** | **14.2237** | **5990.25** |  | **29.6** | **3460.63** | **13.7873** | **8337.93** |
| 17 | 29.0 | 2715.75 | 14.2257 | 7094.47 |  | 31.4 | 3479.45 | 13.7876 | Extended dominance^a^ |
| 16 | 30.7 | 2733.15 | 14.2270 | 13105.38 |  | 33.2 | 3499.62 | 13.7878 | Extended dominance^a^ |
| 15 | 32.5 | 2751.66 | 14.2281 | 17633.04 |  | 35.3 | 3519.78 | 13.7890 | 34620.38 |
| 14 | 34.5 | 2772.84 | 14.2292 | 18463.72 |  | 37.5 | 3544.88 | 13.7893 | 84191.97 |
| 13 | 36.7 | 2796.94 | 14.2301 | 27552.90 |  | 40.0 | 3572.28 | 13.7894 | 169272.69 |
| 12 | 39.0 | 2825.01 | 14.2303 | 144962.55 |  | 42.6 | 3604.48 | 13.7886 | Dominated^b^ |
| 11 | 41.6 | 2857.65 | 14.2299 | Dominated^b^ |  | 45.5 | 3643.49 | 13.7866 | Dominated^b^ |
| 10 | 44.4 | 2893.75 | 14.2293 | Dominated^b^ |  | 48.7 | 3686.78 | 13.7843 | Dominated^b^ |

ASCVD: atherosclerotic cardiovascular disease; QALYs: quality-adjusted life year; ICER: incremental cost-effectiveness ratio.

^a^ Extended dominance indicates larger ICER than a more effective strategy.

^b^ Dominated indicates higher cost and lower QALYs than the comparator.

## Table S8. Cost-utility of ASCVD threshold strategies for hard ASCVD model by sex

| ASCVD risk threshold (%) | Female | | | |  | Male | | | |
| --- | --- | --- | --- | --- | --- | --- | --- | --- | --- |
|  | Statin  eligible (%) | Costs  (2019 US$) | QALYs | ICER  (US$/QALY) |  | Statin eligible (%) | Costs  (2019 US$) | QALYs | ICER  (US$/QALY) |
| No treatment | 0 | 2542.53 | 14.1542 | Ref |  | 0 | 3266.03 | 13.7297 | Ref |
| 15 | 17.5 | 2653.11 | 14.1986 | Extended dominance^a^ |  | 21.6 | 3407.71 | 13.7725 | Extended dominance^a^ |
| 14 | 19.1 | 2657.99 | 14.2036 | 956.76 |  | 23.4 | 3417.26 | 13.7767 | 3220.45 |
| 13 | 20.7 | 2666.00 | 14.2079 | 1866.70 |  | 25.4 | 3430.60 | 13.7801 | 3855.44 |
| 12 | 22.5 | 2675.47 | 14.2126 | 2275.21 |  | 27.6 | 3446.22 | 13.7838 | 4277.29 |
| 11 | 24.5 | 2688.68 | 14.2167 | 3188.24 |  | 30.0 | 3467.19 | 13.7863 | 8466.86 |
| **10** | **26.8** | **2704.13** | **14.2208** | **3762.42** |  | **32.7** | **3492.69** | **13.7883** | **12424.39** |
| 9 | 29.3 | 2725.58 | 14.2241 | 6472.68 |  | 35.8 | 3526.15 | 13.7890 | 51407.70 |
| 8 | 32.1 | 2753.06 | 14.2264 | 12170.51 |  | 39.3 | 3565.60 | 13.7891 | 299944.56 |
| 7.5 | 33.7 | 2769.65 | 14.2274 | 17068.80 |  | 41.2 | 3589.36 | 13.7884 | Dominated^b^ |
| 7 | 35.4 | 2788.48 | 14.2280 | 28715.49 |  | 43.2 | 3616.04 | 13.7875 | Dominated^b^ |
| 6 | 39.3 | 2831.72 | 14.2292 | 38249.01 |  | 47.9 | 3676.77 | 13.7850 | Dominated^b^ |
| 5 | 43.7 | 2888.72 | 14.2282 | Dominated^b^ |  | 53.4 | 3755.07 | 13.7797 | Dominated^b^ |

ASCVD: atherosclerotic cardiovascular disease; QALYs: quality-adjusted life year; ICER: incremental cost-effectiveness ratio.

^a^ Extended dominance indicates larger ICER than a more effective strategy.

^b^ Dominated indicates higher cost and lower QALYs than the comparator.
